# Therapeutic regulation of gut microbiota in gastric cancer: mechanisms, strategies, and clinical prospects

**DOI:** 10.3389/fmicb.2026.1862223

**Published:** 2026-08-24

**Authors:** Zicheng Liu, Mingyue Bai, lichao Han, Zeguang Li

**Affiliations:** 1 The First Clinical Medical College, Heilongjiang University of Chinese Medicine, Harbin, Heilongjiang, China; 2 Zhuhai Hospital of the Guangdong Provincial Hospital of Traditional Chinese Medicine, Zhuhai, Guangdong, China; 3 The First Affiliated Hospital of Heilongjiang University of Chinese Medicine, Harbin, Heilongjiang, China

**Keywords:** gastric cancer, gut microbiota, *Helicobacter pylori*, microbiota imbalance, probiotics

## Abstract

Gastric cancer (GC) is a highly prevalent malignancy associated with substantial mortality worldwide. Dysbiosis of the gut microbiota is closely linked to the pathogenesis, clinical characteristics, and therapeutic responses of GC, making it a central focus of research in the field of tumor microecology. However, existing reviews mainly focus on microbial compositional features, individual mechanisms, or specific microbiota-based interventions, while lacking an integrated theoretical framework that incorporates *Helicobacter pylori* (HP) infection, multi-layer tissue injury, and systematic clinical strategies. Therefore, this review establishes a three-dimensional integrated framework encompassing etiology, injury, and intervention to provide a critical and comprehensive analysis. In the etiological dimension, we systematically summarize GC-associated gut microbiota alterations and their clinical relevance. In the injury dimension, we integrate multiple pathogenic processes, including mucosal barrier disruption, chronic inflammation, carcinogenic metabolite accumulation, epigenetic damage, tumor immunosuppression, and cancer-promoting signaling pathways, to elucidate the potential mechanisms by which the gut microbiota contributes to GC initiation and progression. In the intervention dimension, we systematically evaluate microbiota-modulating strategies, including probiotics, fecal microbiota transplantation, antibiotics, and microbial metabolites, and comparatively assess the evidence levels, advantages, and clinical limitations of these approaches. By integrating clinical studies, experimental models, and translational medical evidence, this review aims to establish a systematic and critical theoretical framework for GC microecological regulation, elucidate the potential and limitations of gut microbiota-based approaches in GC prevention and treatment, and provide new perspectives for the development of precision microecological intervention strategies in the future.

## 1 Introduction

GC remains a major global public health burden. While its incidence and mortality have declined in some regions, GC still ranks among the leading causes of cancer-related death worldwide, with a particularly high disease burden in Asia (Yang et al., 2023). Despite advances in early diagnosis and therapeutic efficacy, GC is still associated with high mortality and suboptimal treatment outcomes, posing a persistent global public health challenge. Therefore, identifying novel preventive and therapeutic strategies is critical to reduce GC incidence and mortality.

With the progressive advancement of gut microbiota research, numerous studies have validated the pivotal role of intestinal microbes in tumor initiation and progression. Gut microbiota regulates the tumor microenvironment and participates in the occurrence, progression, and metastasis of tumors either directly or indirectly via microbial metabolites and host–microbe interactions. The correlation between gut microbiota and GC has emerged as a novel and promising research hotspot in GC research. However, despite the rapid advances in this field in recent years, the precise biological significance of gut microbiota alterations in GC development remains incompletely understood. A critical scientific question is whether gut microbiota dysbiosis serves as a direct driver of gastric carcinogenesis or merely reflects secondary ecological alterations during tumor initiation and progression. Current population-based studies have primarily demonstrated associations between microbiota profiles and GC phenotypes. Although mechanistic evidence from animal models and experimental studies suggests that certain microbial factors may contribute to gastric carcinogenesis, their causal roles in human GC remain to be fully validated. Therefore, the role of the gut microbiota in GC should not be simply interpreted as a universal carcinogenic factor but rather understood within a more complex, dynamic, and context-dependent framework.

At present, numerous reviews have summarized the relationship between the gut microbiota and GC; however, most have focused primarily on individual bacterial species, specific signaling pathways, or potential therapeutic strategies, while lacking systematic integration of these findings into a unified theoretical framework. Moreover, a comprehensive research framework for evaluating relevant mechanisms according to different levels of evidence remains lacking. Therefore, it is essential to further distinguish mechanisms that are well supported by current evidence from those that remain exploratory. In this review, we propose a novel three-dimensional integrated framework centered on the interconnected dimensions of etiology, injury, and intervention as the overarching narrative structure. Moving beyond the conventional perspective that considers microbiota dysbiosis solely as an independent carcinogenic factor, we analyze the dynamic and multifactorial processes underlying microbiota alterations in GC, including their contributing factors, key mechanisms involved in tumor initiation and progression, and current microbiota-targeted therapeutic strategies. By integrating evidence from microbial ecology, host–microbiota interactions, and clinical translation, and critically evaluating the limitations and challenges of existing studies, this review aims to provide a more comprehensive and objective understanding of the potential and constraints of gut microbiota-based strategies for GC prevention and treatment. Furthermore, it offers a theoretical foundation for the development of future precision intervention approaches targeting the tumor microenvironment and gut microecology.

## 2 Changes in gut microbiota in GC

The initiation and progression of GC represent a complex process involving genetic susceptibility, environmental exposures, immune regulation, and disruption of microecological homeostasis. In recent years, advances in metagenomic sequencing and multi-omics technologies have revealed significant alterations in the composition and functional capacity of the gut microbiota in patients with GC, suggesting that microecological dysregulation may serve as a critical link between environmental factors and gastric carcinogenesis. Based on the “three-dimensional regulatory framework” proposed in this review, this section first systematically examines the characteristics and determinants of GC-associated intestinal microecological alterations from the perspective of the “etiology dimension.” By comparing gut microbiota profiles between healthy individuals and patients with GC, we first characterize the fundamental features of GC-related microecological dysbiosis. Subsequently, we explore the roles of key regulatory factors, including host genetic background, dietary patterns, and environmental exposures, in shaping the “microbiota–GC axis,” thereby elucidating the heterogeneity of microbial alterations across different study cohorts. On this basis, we analyze the association between microbiota dysbiosis and GC risk and further summarize the relationships between microbial changes, clinicopathological characteristics, and disease progression. Notably, most existing studies primarily demonstrate associations between microbiota alterations and GC, whereas whether microbiota dysbiosis directly drives gastric carcinogenesis remains to be determined through longitudinal cohort studies and mechanistic investigations. The discussion in this section provides a theoretical foundation for subsequent analyses of microbiota-mediated inflammatory, immune, metabolic, and molecular mechanisms from the “injury dimension,” as well as for the exploration of precision microecological therapeutic strategies from the “intervention dimension.”

### 2.1 Differences in gut microbiota composition between Normal stomach and GC patients

**Figure 1:**
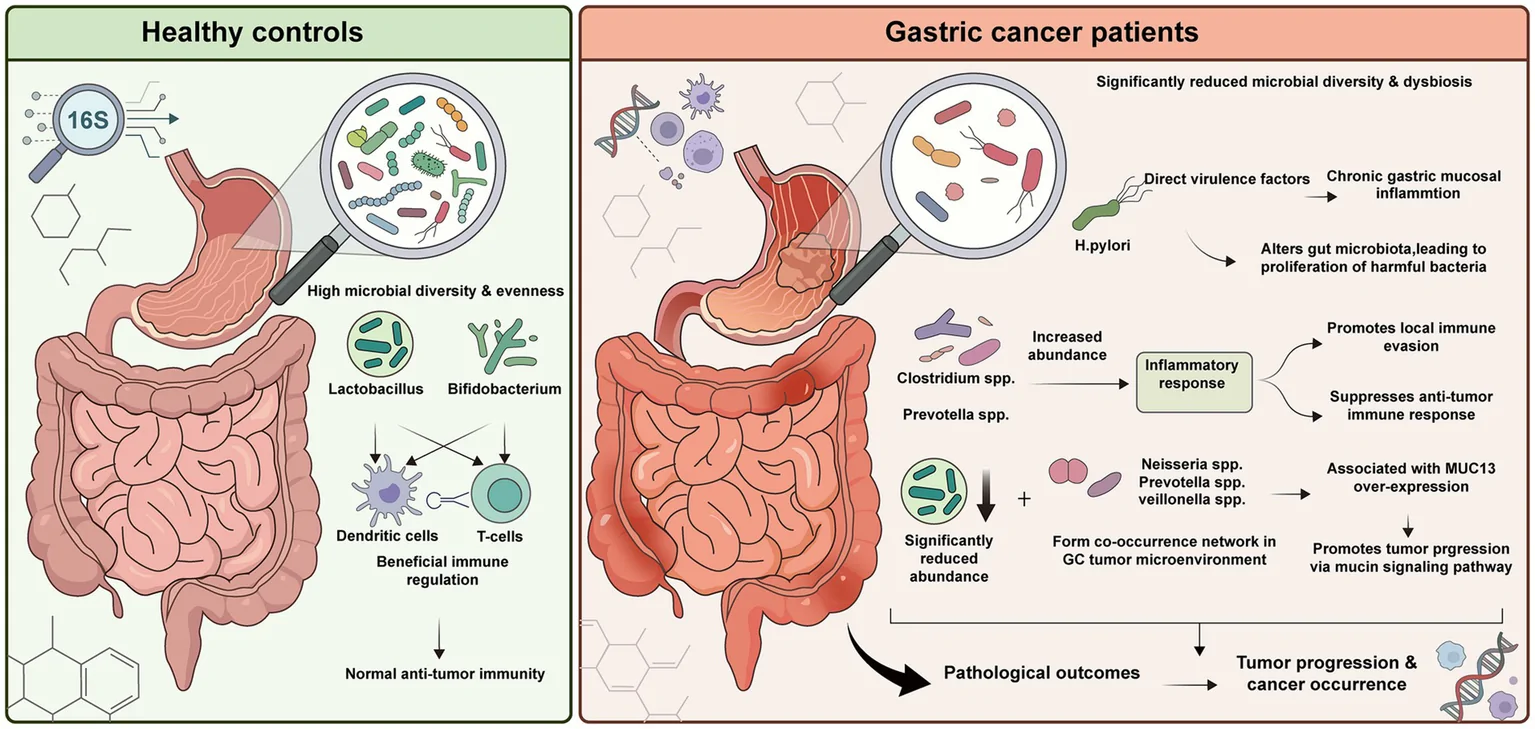
Differences in gut microbiota composition between healthy individuals and GC patients.

The gut microbiota of the GC patients had significantly less diversity and structural imbalance compared to healthy controls (Figure 1). Recent 16S rRNA gene sequencing analyses have clarified the gut microbiota characteristics of GC patients, enabling accurate evaluation of microbial diversity and population groups. Research findings suggest that the *α*-diversity (species richness and evenness) in GC patients is significantly lower than in healthy controls, hence indicating a depletion of microbial species and a non-even distribution (Dong et al., 2019). Moreover, the *β*-diversity (microbial community structure) in GC patients notably differs from that in healthy individuals, especially in advanced GC (Chen C. et al., 2025). The gut microbiota composition differs drastically between healthy individuals and GC patients. The healthy gut microbiota is dominated by anaerobic bacteria, mainly lactic acid bacteria and bifidobacteria, which participate in host immune regulation and maintain mucosal homeostasis. On the contrary, the gut microbiota of GC patients has reduced diversity and enriched pathogenic bacteria. Specifically, GC patients is enriched in *Clostridium* and *Prevotella*, which are closely associated with intestinal inflammatory responses and may facilitate GC carcinogenesis by inducing local immune escape or blocking the anti-tumor immune responses (Zhang J. et al., 2024; Zhang R. et al., 2024). Conversely, the abundance of beneficial bacteria, including lactic acid bacteria, is markedly decreased in GC patients, which may mediate immune tolerance and hinder anti-tumor immunity (Lazăr et al., 2025). Specific bacterial taxa are critical drivers of GC development. To date, HP infection is the most dominant and essential pathogenic factor for GC. HP not only induces chronic gastric mucosal inflammation via virulence factors such as cytotoxin-associated gene A (CagA) protein but also reshapes gastric and gut microbiota composition by promoting pathogenic bacterial proliferation, thereby accelerating GC progression (Sharafutdinov et al., 2023). Furthermore, studies have identified a co-occurrence network of *Neisseria*, *Prevotella*, and *Veillonella* in the GC tumor microenvironment, which is correlated with MUC13 overexpression, suggesting that these bacteria may promote tumor progression through mucin-mediated signaling pathways (Oosterlinck et al., 2023). Investigations on the dysregulation of key intestinal bacterial taxa in GC patients help clarify GC pathogenesis and identify potential microbial therapeutic targets.

A substantial number of studies based on 16S rRNA and metagenomic sequencing have demonstrated that intestinal microbial homeostasis is disrupted in patients with GC, and that their microbial composition differs significantly from that of healthy individuals. However, inconsistencies across studies remain notable. The pronounced microbial alterations observed in populations from high-incidence regions such as China and South Korea have not been consistently replicated in studies from low-incidence regions, including Europe and the United States. In particular, several European and American studies have failed to identify significant differences in gut microbial composition between GC patients and healthy controls (Zhang et al., 2024b). This inconsistency may arise from a combination of biological and methodological factors. First, population-specific host characteristics across different regions, including genetic background, dietary patterns, lifestyle behaviors, and environmental exposures, may substantially influence microbial ecosystem composition and subsequently modulate host–microbiota interactions. For instance, in regions with a high incidence of GC, high-salt diets and specific food preservation practices are more prevalent. These factors may contribute to GC risk by altering intestinal microbial composition, promoting inflammatory responses, and affecting the gastric mucosal microenvironment (Wu et al., 2022). Second, variations in study design and detection methodologies represent important contributors to inconsistent findings. Currently, sample sources differ substantially among studies, including fecal and gastric tissue samples, and microbial ecological profiles derived from different sampling sites may vary considerably. Fecal microbiota primarily reflects overall intestinal microbial alterations, whereas gastric tissue microbiota more directly represents the microbial composition within the local tumor microenvironment. Moreover, differences in data processing workflows, bioinformatics pipelines, and statistical analysis strategies may further influence the microbial profiles identified in different studies.

In addition, current research on the GC microbiome is generally limited by a key methodological constraint: most studies are cross-sectional and based on association analyses. Such designs can identify differences in microbial composition between patients with GC and non-cancer controls at a specific time point (e.g., after diagnosis), but they primarily provide correlational evidence rather than causal inference (Bessède and Mégraud, 2022; Gao et al., 2026). For example, studies have reported increased abundances of Lactobacillus, Streptococcus, and other bacterial taxa in GC tissues, whereas Helicobacter is more abundant in normal gastric tissues (Dai et al., 2021). This type of research primarily reveals associations between microbiota alterations and GC phenotypes but cannot directly establish causal relationships. Therefore, future studies should integrate long-term longitudinal cohort studies, multi-omics approaches, and functional validation experiments. Transitioning from the “description of microbiota differences” to the “investigation of microbiota functions and causal mechanisms” will facilitate a more accurate understanding of the actual role of the gut microbiota in GC initiation and progression.

### 2.2 Host genetics, diet, and environmental factors: non-negligible regulators of the microbiota–GC axis

Microbiota composition is not an independent exposure variable but rather a downstream phenotype shaped by host genetic background, long-term dietary patterns, and cumulative environmental exposures. Host genomic polymorphisms represent a key intrinsic determinant underlying interindividual differences in GC susceptibility despite similar microbiota exposures. These polymorphisms can be broadly categorized into two major groups: immune response-related gene polymorphisms and gastric acid secretion-related gene polymorphisms. Immune response-related gene polymorphisms: Functional polymorphic variants exist in genes encoding key inflammatory mediators, including IL-1β, TNF-*α*, TLR4, and IL-8. These genetic variations influence the magnitude of host inflammatory responses to HP and lipopolysaccharide (LPS) derived from specific Gram-negative bacteria within the commensal microbiota. Even under comparable bacterial colonization levels, individuals carrying high-inflammatory-response genotypes exhibit enhanced gastric mucosal neutrophil infiltration, increased accumulation of reactive oxygen and nitrogen species, and consequently accelerated DNA damage and epithelial metaplasia. Gastric acid secretion-related gene polymorphisms: Polymorphisms in ATP4A and ATP4B, which encode gastric proton pump components, directly influence gastric pH and mucus layer integrity, thereby shaping microbial selection through an “acidic filtering” process. A low-acid environment favors the colonization of opportunistic pathogens such as *Veillonella* and *Neisseria*, whereas a highly acidic environment tends to select for acid-resistant beneficial bacteria, including lactic acid bacteria. This genetically determined microenvironmental context enables individuals with similar microbiota exposure profiles to develop substantially different microbial compositions and functional outputs, such as metabolite profiles (Fu et al., 2021).

Diet represents a key external factor that dynamically shapes microbiota composition. Different dietary patterns can modulate microbial abundance, alter microbial metabolite profiles, and directly influence interactions between the microbiota and gastric mucosa. A high-salt diet is a characteristic dietary pattern observed in regions with a high incidence of GC. Its effects extend beyond direct damage to the gastric mucosal epithelium; high salt intake may also selectively promote the colonization advantage of HP and certain pro-inflammatory commensal bacteria by increasing gastric osmotic pressure and disrupting epithelial barrier integrity, while simultaneously reducing the abundance of beneficial acid-producing bacteria such as *Bifidobacterium* (Loh et al., 2023). Conversely, a high-fiber diet may exert anti-inflammatory effects by increasing the production of short-chain fatty acids (SCFAs), particularly butyrate, which can inhibit histone deacetylase (HDAC) activity and partially counteract genetically predisposed excessive inflammatory responses (Chen and Vitetta, 2018).

The history of antibiotic exposure, early-life microbiota colonization patterns, familial aggregation of HP infection, and regional healthcare conditions collectively constitute an individual’s “environmental microbiota exposure profile.” These factors shape the initial ecological niches established by the early-life microbiota and influence the resilience and adaptability of the microbial ecosystem in adulthood when confronted with external perturbations, such as chemotherapy and antibiotic exposure. Differences in conclusions regarding microbiota–GC associations between East Asian and European/American populations may largely be attributed to systematic variations in antibiotic exposure patterns (e.g., the more widespread use of HP eradication strategies in East Asia), dietary patterns (high-salt preserved foods versus high-fiber Western diets), and population-specific genetic backgrounds. The central implication of these three regulatory factors is that the microbiota should not be regarded as an independent causal determinant but rather as a dynamic output of the interaction between the host genetic “immune-inflammatory tone” and the “continuous selection pressure” imposed by diet and environmental exposures. Therefore, at the mechanistic level, future longitudinal cohort studies should simultaneously incorporate genome-wide genetic risk scores, standardized dietary frequency questionnaires, and cumulative antibiotic exposure indices; otherwise, temporal causal relationships may be obscured by baseline interindividual heterogeneity.

### 2.3 Microbial imbalance and GC risk

Increasing studies have focused on the correlation between gut microbiota dysbiosis and GC risk. Microbiota dysbiosis refers to abnormal alterations in the diversity, abundance, and functional composition of microbial communities, and gut microbiota dysbiosis is tightly linked to the initiation and progression of GC. Clinical studies have demonstrated that gastric mucosal tissues from AGC patients exhibit lower microbial *α*-diversity and significant taxonomic dysbiosis (characterized by selective enrichment and depletion of specific bacterial species) compared with tissues from chronic gastritis patients and healthy individuals (Coker et al., 2018). Microbiota dysbiosis facilitates the production of carcinogenic substances such as nitrites and amines, which trigger persistent chronic inflammation and continuous stimulation of precancerous gastric lesions, ultimately driving gastric mucosal carcinogenesis. The depletion of oral and gastric commensal bacteria including Streptococcus and Prevotella impairs nitrate reduction and nitrite degradation, allowing nitrites to bind with amines and form highly carcinogenic N-nitroso compounds (Xia et al., 2025). Although HP infection is a well-established GC risk factor, its carcinogenic effect depends largely on the overall composition of gut microbiota. HP infection reduces gastric acid secretion, and creates a niche for the colonization of exogenous oral and intestinal bacteria, further exacerbating gastric mucosal inflammation and atrophy (Bessède and Mégraud, 2022). Microbiota dysbiosis-induced immunosuppression and immune escape support the survival and proliferation of GC cells. Pathogenic bacteria such as *Streptococcus anginosus* can remodel host immune responses, impair tumor immune surveillance, and promote gastric carcinogenesis (Yuan et al., 2024). Reduced gastric acid secretion also leads to the overproliferation of intestinal commensal bacteria, including *Escherichia coli* and *Proteobacteria* in the stomach. Bacterial components such as LPS trigger inflammatory cascades and promote the secretion of pro-inflammatory cytokines, which stimulate cell proliferation, inhibit cell apoptosis, and facilitate tumor formation (Png et al., 2022).

### 2.4 Association between changes in gut microbiota and clinicopathological features

The composition of gut microbiota is closely correlated with the clinical stage, pathological type, and prognosis of GC, with stage-specific bacterial biomarkers identified during GC progression. Early-stage GC patients exhibit elevated abundance of *Collinsella* and Lachnospiraceae, which may be related to postoperative microbial remodeling (Chen et al., 2022). In severe cases of GC, there is a significant increase in *Escherichia*-*Shigella* and *Klebsiella*. These microbes increases the N-acetylcadaverine in the microenvironment. This compound is thought to worsen prognosis through various pro-inflammatory effects (Zou et al., 2025). For metastatic GC, chemotherapy-induced cachexia and muscle wasting are strongly associated with the enrichment of *Enterococcus faecalis* and enterotoxigenic *Bacteroides fragilis* (Wu J. et al., 2024). Research indicates that stage-specific dysbiosis of the gut microbiota occurs with GC occurrence and development. The *α*-diversity of gut microbiota decreases and *β*-diversity alters significantly in the progression of superficial gastritis (SG) and atrophic gastritis (AG) to gastric mucosal dysplasia (GMD) and advanced GC. Lactobacillus and other symbiotic bacteria take dominance in the early stages (SG/AG), where their metabolites of folic acid and methionine may postpone carcinogenesis by maintaining a barrier function for the mucosa (Liu et al., 2020). In this stage, a reduction in Prevotellaceae, Ruminococcaceae, and other short-chain fatty acid (SCFA)producing bacteria may be correlated with an immune microenvironment imbalance (Pan et al., 2021). In advanced GC, the microbial community is characterized by an enrichment of *Proteobacteria*, particularly Proteus species, along with an increased abundance of *Streptococcus anginosus*. These microbial alterations may promote tumor progression by enhancing inflammatory responses and suppressing CD8^+^ T cell function (Yuan et al., 2024). Changes in gut microbiota may modify response of GC patients to chemotherapy which may alter clinical efficacy and outcome. Research indicates a positive association between the abundance of Lactobacillus and the effectiveness of PD-1/programmed cell death-ligand 1(PD-L1) inhibitors (Marashi et al., 2024). Moreover, the immunotherapy is better in microbial sub-type (MS1) that is high on butyrate-producing bacteria. Poor responses to adjuvant chemotherapy are microbial sub-type MS3 which is high on *Proteobacteria* (Wang et al., 2024).

In summary, the intestinal microecology undergoes dynamic, stage-specific evolution during the initiation and progression of GC. These alterations are not merely characterized by changes in the abundance of individual microbial taxa but also involve continuous ecological remodeling processes, including reduced microbial diversity, depletion of beneficial commensal bacteria, and expansion of pro-inflammatory microbial populations, as summarized in Table 1. It should be noted that stage-specific microbial markers do not currently enable standardized diagnostic applications across populations. Significant differences exist in enriched microbial taxa at the same disease stage among Asian, European, and American cohorts, and no uniform or reproducible non-invasive microbial model for cancer staging has been established. These limitations are further compounded by confounding factors such as regional dietary patterns, HP infection prevalence, clinical treatment history, and differences in sampling sites (e.g., fecal versus gastric mucosal samples). As a result, most existing predictive models are derived from single-center training datasets and lack validation in independent external cohorts, indicating insufficient level I evidence.

**Table 1:** Stage-specific alterations of gut microbiota during GC progression and their potential implications.

Disease stage	Representative microbial alterations	Potential biological implications	Current evidence level
SG/ AG	Increased abundance of *Lactobacillus* and other commensal bacteria	Maintain mucosal homeostasis and regulate inflammation	Relatively consistent but population-dependent
GMD / intestinal metaplasia	Decreased abundance of SCFA-producing bacteria (e.g., Prevotellaceae and Ruminococcaceae)	Impaired immune regulation and mucosal protection	Moderate evidence, mainly from observational studies
Early GC	Enrichment of *Collinsella* and Lachnospiraceae	May reflect microbiota remodeling during tumor development	Preliminary evidence requiring further validation
Advanced GC	Increased abundance of *Proteobacteria*, *Proteus*, *Escherichia*-*Shigella*, *Klebsiella*, and *Streptococcus anginosus*	Promote inflammation and immune dysregulation	Supported by multiple studies
Metastatic GC	Increased abundance of *Enterococcus faecalis* and enterotoxigenic *Bacteroides fragilis*	Associated with systemic inflammation and disease progression	Emerging evidence

Across multiple lines of evidence, reduced microbial diversity and enrichment of pro-inflammatory and pathogenic bacteria appear to be consistent features throughout the course of GC. Moreover, HP infection and microbial dysbiosis may synergistically contribute to gastric carcinogenesis, supported by relatively robust mechanistic evidence. However, due to substantial inter-population heterogeneity, microbial markers can only serve as auxiliary stratification tools and are not suitable as standalone biomarkers for early non-invasive diagnosis of GC. Current research is largely based on cross-sectional correlation analyses, and the absence of prospective longitudinal cohorts capable of distinguishing whether microbial dysbiosis is a cause or consequence of carcinogenesis represents a major limitation in this field.

## 3 Mechanistic link between gut microbiota and GC

The mechanisms underlying the interaction between the gut microbiota and GC have become a major focus of current cancer research. Building upon the preceding analysis of GC-associated intestinal microecological alterations and their determinants, this section further explores the potential mechanisms by which the gut microbiota contributes to GC initiation and progression from the perspective of the “injury dimension” within the “three-dimensional regulatory framework” proposed in this review. Within this framework, we integrate microbiota-mediated pathological processes associated with GC into six interconnected injury pathways: immune microenvironment remodeling, chronic inflammatory damage, microbial metabolic dysregulation, disruption of the gastric mucosal barrier, DNA damage and cellular malignant transformation, and regulation of key molecular signaling networks. Among these processes, immune dysregulation and chronic inflammation may represent initial events through which microbiota abnormalities disrupt gastric mucosal homeostasis. Subsequently, metabolic alterations and barrier dysfunction further facilitate the establishment of a tumor-promoting microenvironment. Persistent oxidative stress, DNA damage, and aberrant activation of signaling pathways promote malignant cellular transformation and tumor progression. These processes do not occur independently but instead constitute a dynamic feedback network involving inflammatory mediators, immune cells, microbial metabolites, and host signaling pathways, thereby collectively driving the disruption of microbiota–host interactions. Therefore, this section extends beyond a simple summary of specific molecular pathways associated with individual microbial alterations and aims to establish a multilevel, dynamic mechanistic model illustrating how gut microbiota dysbiosis progresses from early ecological disturbance to host tissue injury and ultimately contributes to GC development. This mechanistic framework also provides a theoretical foundation for subsequent discussions on microbiota-targeted therapeutic strategies directed at distinct injury pathways.

### 3.1 Interaction between microbiota and the immune system

**Figure 2:**
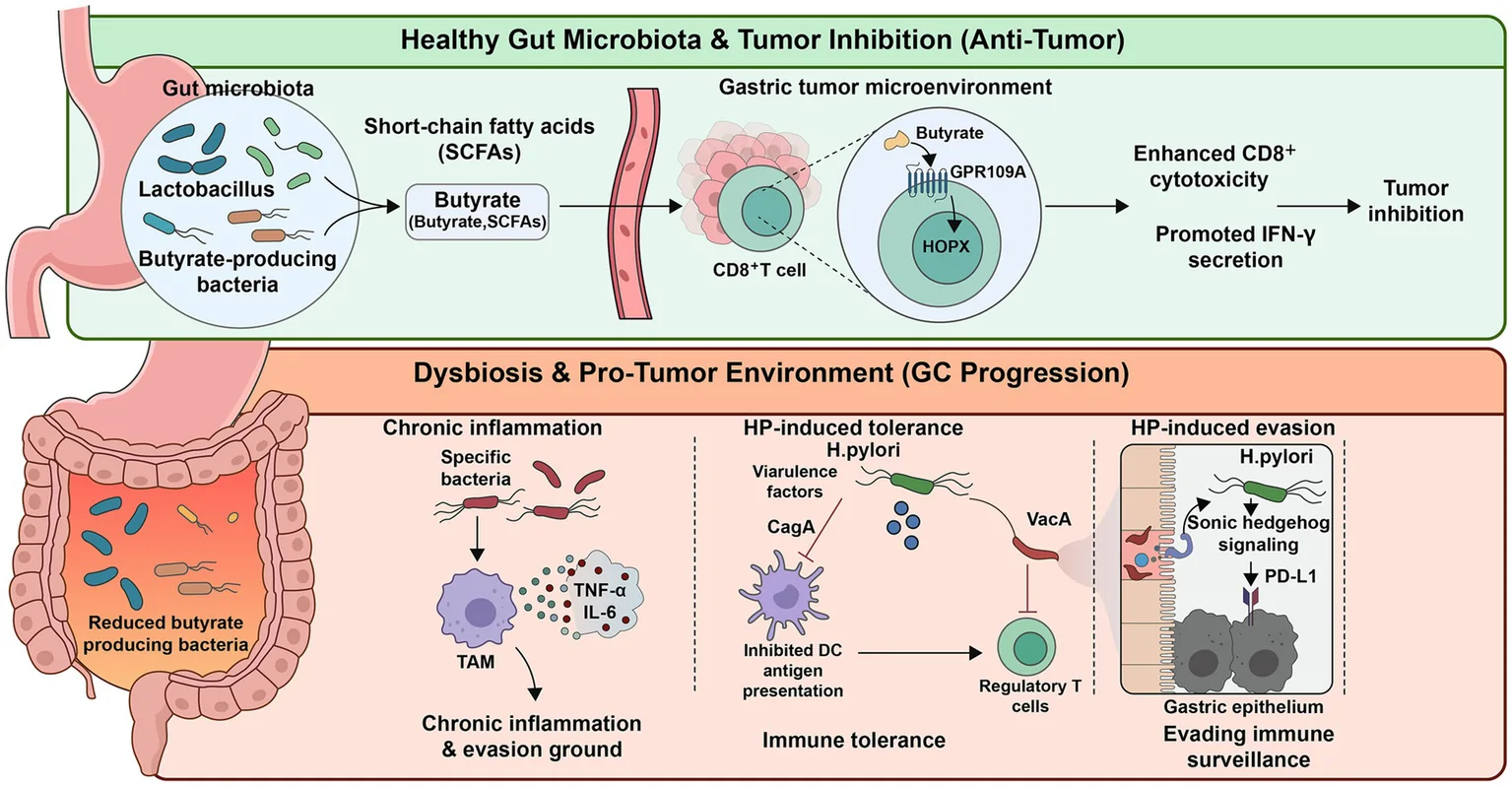
Interaction between gut microbiota and immune system in GC development. Gut microbiota contributes to GC development by regulating metabolite production, immune cell function, and immune evasion. Beneficial commensal bacteria produce SCFAs, among which butyrate enhances CD8^+^ T-cell cytotoxicity and promotes IFN-γ secretion through activation of the GPR109A/HOPX signaling pathway, thereby exerting antitumor effects. Conversely, gut microbial dysbiosis, characterized by a reduction in butyrate-producing bacteria, can activate TAMs, increase the release of pro-inflammatory cytokines such as TNF-α and IL-6, and promote chronic inflammation. Moreover, HP virulence factors CagA and VacA impair DC antigen presentation, promote Treg expansion, and induce immune tolerance. HP infection can also upregulate PD-L1 expression through the Hedgehog signaling pathway, thereby facilitating immune escape of tumor cells. In addition, gut microbial composition influences systemic antitumor immunity and responses to immunotherapy. *Lactobacillus* species enhance Th1 and CD8^+^ T-cell activation, whereas certain microbial populations induce Foxp3^+^ Treg expansion and establish an immunosuppressive tumor microenvironment.

Gut microbiota modulates the gastrointestinal immune microenvironment locally and regulates systemic immune function remotely, enabling tumor cells to evade immune detection and promoting carcinogenesis (Figure 2). The microbiota induces immune responses in GC by means of SCFA, e.g., butyrate. SCFAs can regulate immune cell function by inhibiting HDAC activity (Kibbie et al., 2021). A landmark study demonstrated that butyrate significantly enhances the cytotoxic activity of CD8^+^ T cells and promotes interferon-*γ* (IFN-γ) secretion by activating the G protein–coupled receptor 109A (GPR109A) and its downstream HOPX signaling pathway, thereby inhibiting tumor growth. These findings provide strong evidence that microbial metabolites can modulate the immune microenvironment in GC (Yu et al., 2024). An imbalance in microbes, like a decrease in bacteria producing butyrate, may generate an immunosuppressive microenvironment that accelerates tumor development (Guo et al., 2022). Chronic inflammation response often relates to GC. Specific intestinal bacteria or their metabolites can modulate the polarization of TAMs in the gastric mucosa. Activated TAMs can exacerbate local inflammation and create a “fertile soil” for tumorigenesis by secreting pro-inflammatory cytokines (Zhang et al., 2020). However, the regulatory effects of the gut microbiota on tumor-associated macrophages (TAMs) are highly complex, and different microbial strains may exert opposing effects. At present, the specific regulatory network underlying these interactions in GC remains to be fully elucidated. The gut microbiota increases its inhibitory effect by enhancing T-cell activation. This immunosuppressive environment deters the host’s anti-tumor immune response and allows cancer proliferation and metastasis, in part due to the inhibitory effect on anti-tumor T cell functioning. A study shows that CagA and VacA virulence factors of HP weaken CD8 + T cell response due to targeted disruption of antigen presenting function of dendritic cells (DCs), promoting expansion of regulatory T cells (Tregs) and establishing immune tolerance (Huang and Hu, 2025). Moreover, HP induces PD-L1 expression in gastric epithelial cells via the sonic hedgehog signaling pathway, further allowing HP-infected cells to evade immune surveillance and promoting the progression of GC (Holokai et al., 2019). Certain microbiota such as Lactobacillus have been demonstrated to activate Th1 and CD8 + T cells, whereas others such as *Bacteroides fragilis* may induce immune tolerance through signaling that stimulates Foxp3 + Tregs and IL-10 secretion (Fakharian et al., 2022). It is worth noting that negative evidence has also been reported regarding the interaction between the gut microbiota and immunotherapy. For instance, the use of broad-spectrum antibiotics can disrupt intestinal microbial homeostasis, reduce the abundance of beneficial bacteria such as Lactobacillus, and increase the proportion of exhausted CD8^+^ T cells, thereby diminishing the efficacy of PD-1 inhibitors (Ng et al., 2025). These findings further underscore the importance of a balanced intestinal microecology for maintaining effective anti-tumor immune responses.

### 3.2 Chronic inflammation induced by the microbiome

**Figure 3:**
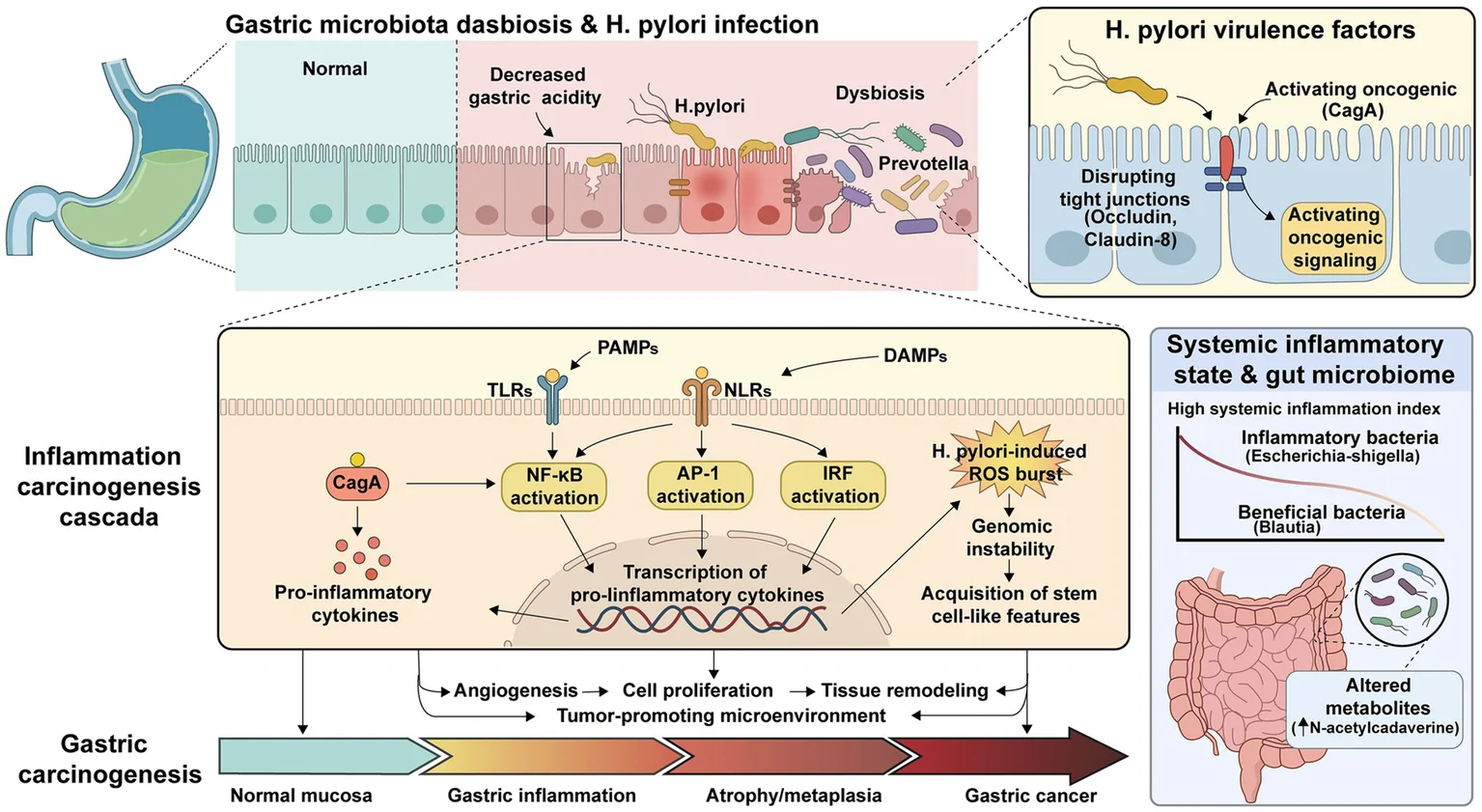
Chronic inflammation induced by gut microbiota imbalance in GC. Gastric microecological dysbiosis and HP infection induce chronic inflammation and contribute to gastric carcinogenesis. PAMPs and DAMPs generated by microbial dysbiosis are recognized by PRRs, including TLRs and NLRs, thereby activating NF-κB, AP-1, and IRF signaling pathways and promoting the secretion of pro-inflammatory cytokines. The HP virulence factor CagA disrupts gastric epithelial tight junctions, activates oncogenic signaling pathways, and induces the expression of inflammatory mediators. Meanwhile, HP-induced ROS accumulation causes genomic instability and promotes the acquisition of stem cell-like characteristics, thereby accelerating gastric carcinogenesis. Under conditions of reduced gastric acidity and microbial imbalance, opportunistic commensal bacteria become abnormally enriched and sustain inflammatory signaling through microbial metabolites, ultimately establishing a tumor-promoting microenvironment. In addition, systemic inflammatory status is closely associated with intestinal microbial composition. The expansion of pro-inflammatory bacteria, depletion of beneficial microbes, and accumulation of abnormal metabolites (e.g., elevated N-acetylcadaverine levels) collectively contribute to inflammation-driven gastric carcinogenesis.

Gut microbiota imbalance, particularly the overgrowth of pathogenic microorganisms, triggers chronic intestinal inflammation and further predisposes individuals to GC development (Figure 3). Microbe-induced inflammatory responses are primarily initiated via pattern recognition receptors (PRRs). Under microbiota dysbiosis, host cell PRRs, including Toll-like receptors (TLRs) and NOD-like receptors (NLRs), recognize pathogen-associated molecular patterns (PAMPs) and damage-associated molecular patterns (DAMPs). The activation of these receptors initiates downstream signaling cascades that ultimately activate multiple transcription factors, such as NF-κB, AP-1, and IRF, thereby promoting the transcription and secretion of pro-inflammatory mediators (Nasr et al., 2020). As a class I human carcinogen, HP initiates the inflammation-carcinogenesis cascade in GC. Its core virulence factor CagA disrupts tight junction proteins (occludin and claudin-8) in gastric epithelial cells and activates oncogenic signaling pathways. Specifically, CagA triggers the NF-κB pathway to induce the secretion of pro-inflammatory cytokines, which directly damage gastric mucosal cells and promote angiogenesis, cell proliferation, and tissue remodeling, forming a pro-tumor microenvironment (Ansari and Yamaoka, 2020). HP infection-induced reactive oxygen species (ROS) further aggravates genomic instability and endows gastric cells with stem cell-like properties, laying a critical foundation for gastric carcinogenesis (Nabavi-Rad et al., 2023). Meanwhile, HP-induced gastric acid reduction and gastric ecological imbalance allow exogenous bacteria to colonize the gastric mucosa, thereby exacerbating local inflammatory responses (Yang et al., 2021). Accumulating studies have demonstrated that oral and intestinal commensal bacteria, such as *Streptococcus*, *Prevotella*, and *Peptostreptococcus*, are significantly enriched in the gastric mucosa of GC patients and individuals with precancerous lesions such as intestinal metaplasia (Barra et al., 2021). These bacteria, or their metabolites such as LPS, may activate host pattern recognition receptors, persistently stimulate pro-inflammatory signaling pathways, and thereby establish a pro-tumorigenic inflammatory microenvironment (Nouri et al., 2023). It should be noted that whether this activation is directly driven by these “follower bacteria” or merely reflects a “passenger” phenomenon associated with tumor-associated microenvironmental changes remains to be clarified through further functional studies. Additionally, gut microbiota dysbiosis is closely correlated with systemic inflammation, which profoundly affects the prognosis of GC patients. Patients with a high systemic inflammatory response index (SIRI) exhibit an elevated abundance of pro-inflammatory bacteria (e.g., *Escherichia*-*Shigella*) and depleted beneficial bacteria (e.g., *Blautia*), accompanied by characteristic metabolic alterations such as increased N-acetyl cadaverine levels. This microbial metabolite–covariate network provides a systematic framework for understanding inflammation-driven carcinogenesis (Zou et al., 2025).

In summary, the evidence supporting chronic inflammation pathways mediated by microbiota alterations discussed in this section is primarily derived from *in vitro* cellular and animal studies. Most clinical studies have only demonstrated associations among microbial abundance, inflammatory markers, and GC. The complete causal chain whereby microbiota dysbiosis induces persistent chronic inflammation and directly promotes carcinogenesis in the human gastric mucosa remains to be established.

### 3.3 Carcinogenic effects of metabolites

**Figure 4:**
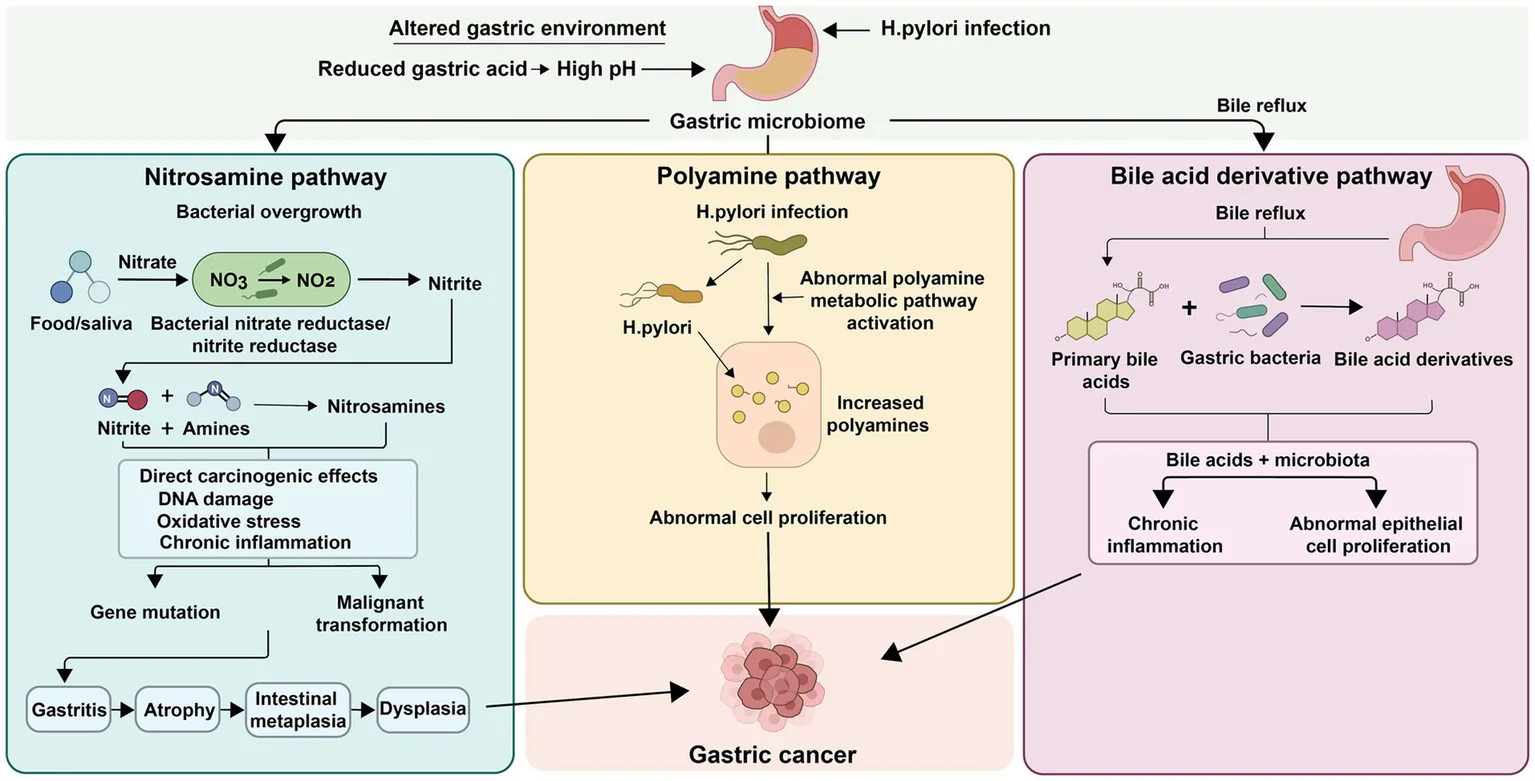
Carcinogenic effects of microbial metabolites in GC development. Alterations in the gastric environment, including reduced gastric acid secretion, increased pH, HP infection, and bile reflux, can disrupt gastric microbial homeostasis and promote the production of various carcinogenic metabolites. Microbiota-derived nitrosamines, polyamines, and bile acid derivatives can impair gastric epithelial cell function and contribute to gastric carcinogenesis through distinct mechanisms. In the nitrosamine pathway, bacteria enriched in the gastric environment convert nitrate into nitrite through nitrate and nitrite reductases, which subsequently react with amines to generate nitrosamines. These compounds induce DNA damage, oxidative stress, and chronic inflammation, thereby promoting genetic mutations and driving the multistep progression of gastric mucosal lesions from gastritis and atrophy to intestinal metaplasia, dysplasia, and ultimately GC. In the polyamine pathway, HP infection disrupts polyamine metabolism, resulting in elevated polyamine levels that promote abnormal proliferation of gastric epithelial cells and provide a metabolic basis for carcinogenesis. Furthermore, bile reflux facilitates interactions between bile acids and gut microbiota, leading to the generation of carcinogenic bile acid derivatives. These metabolites induce chronic inflammation and aberrant epithelial cell proliferation, thereby establishing a tumor-promoting microenvironment and accelerating GC progression.

Gut and gut microbiota facilitate GC progression via producing diverse metabolites that exert direct and indirect carcinogenic effects, as summarized in Figure 4. The production of these metabolites, including organic acids, amines, toxins, gases, and others, represents a key mechanism through which the microbiota influences the function and fate of gastric epithelial cells.

Bacterial nitrate reductase and nitrite reductase are primarily responsible for the generation of nitrosamines, a core class of carcinogenic metabolites. Gastric hypoacidity and elevated intragastric pH reshape the gastric microenvironment, enabling the colonization and proliferation of oral and intestinal bacteria in the stomach. These bacteria convert nitrates from food and saliva into nitrites, which further react with amines to form carcinogenic nitrosamines (Ferreira et al., 2018). Nitrosamines not only directly induce DNA damage but also aggravate oxidative stress and chronic inflammation, trigger oncogenic gene mutations, and promote malignant cell transformation. Additionally, they drive the progressive pathological evolution of gastric tissues, namely the sequential progression from gastritis and gastric atrophy to intestinal metaplasia, dysplasia, and ultimately GC (Zhang et al., 2025).

Polyamines represent another vital category of carcinogenic metabolites that regulate cell proliferation and differentiation. While physiological levels of polyamines maintain normal cellular activities, excessive polyamine accumulation drives aberrant cell proliferation and tumorigenesis. Multiple studies have confirmed significant polyamine accumulation in HP-infected gastric mucosa, and the abnormal activation of polyamine metabolic pathways serves as a key molecular link between HP infection and gastric carcinogenesis (Zhang and Au, 2017; Gobert et al., 2025).

The microbial metabolism of primary bile acids by intestinal bacteria generates a variety of bile acid derivatives. These derivatives play complex and essential roles in both physiological and pathological processes within the gastrointestinal tract. Although bile acids have historically been regarded primarily as digestive agents, increasing evidence suggests broader biological functions. Bile acids can modulate the gastric microbiome and, under certain pathological conditions, may contribute to GC development. Studies have shown that reflux of bile acids into the stomach enables interactions with the gut microbiota. These interactions may shift the microbial community toward a pro-inflammatory state, thereby promoting chronic inflammation, abnormal epithelial cell proliferation, and ultimately GC progression (Huang et al., 2022; Jin et al., 2022; Wang et al., 2022). In turn, GC progression reciprocally reshapes gut microbiota composition, enriching bacteria capable of synthesizing secondary bile acids and thereby boosting the generation of carcinogenic bile acid derivatives (Lee et al., 2020). Consistently, microbial pathways responsible for secondary bile acid synthesis are markedly enriched in GC patients (Gunathilake et al., 2020).

It should be noted that the carcinogenic effects of various microbial metabolites have been primarily demonstrated *in vitro* and animal models, whereas direct clinical evidence supporting their independent capacity to induce malignant transformation of human gastric epithelial cells remains limited.

### 3.4 Imbalance of the microbiota and disruption of the gastric mucosal barrier

**Figure 5:**
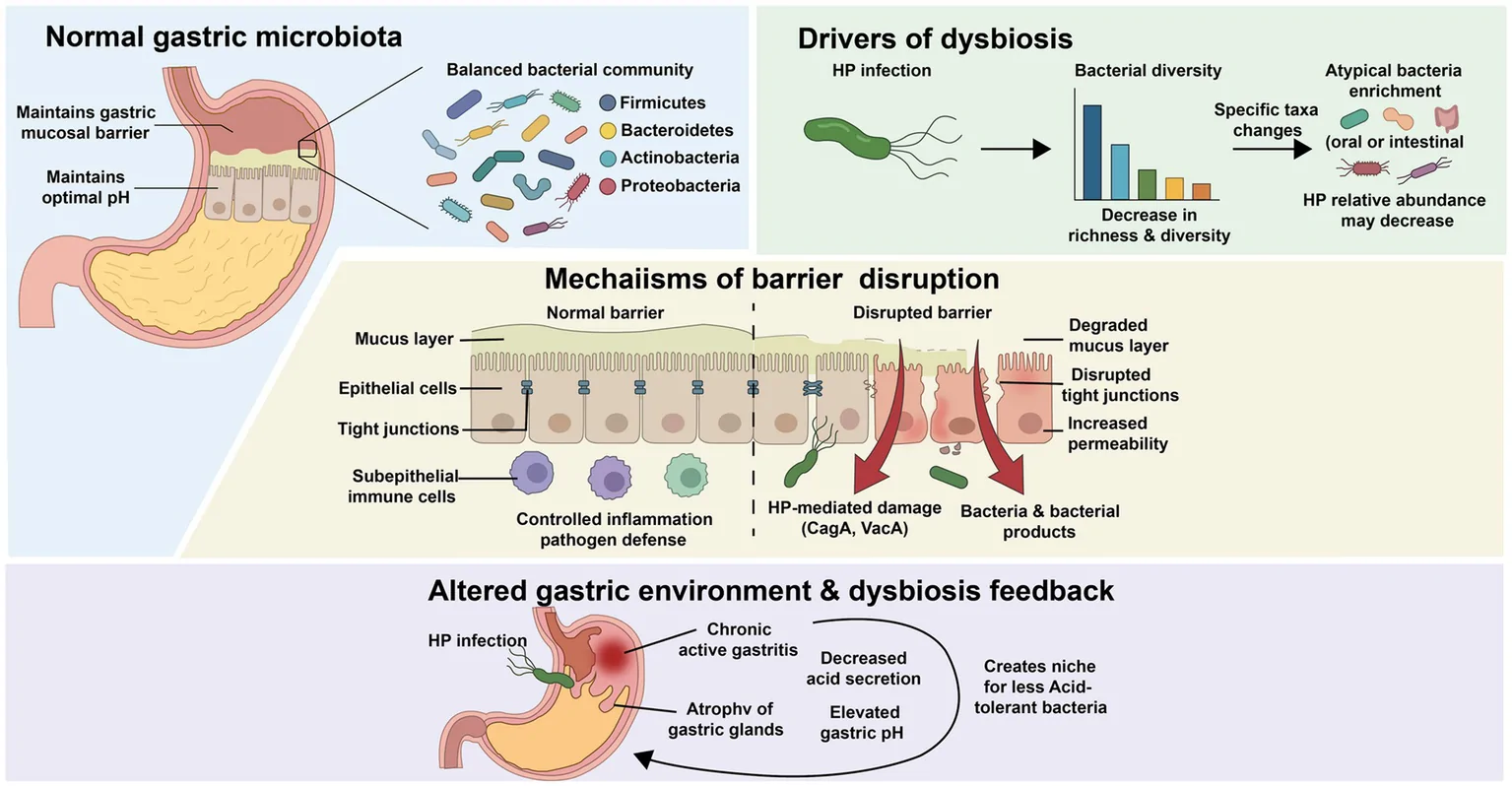
Gut microbiota imbalance and its role in disruption of gastric mucosal barrier in GC. The normal gut microbiota consists primarily of *Firmicutes*, *Bacteroidetes*, *Actinobacteria*, and *Proteobacteria*, which contribute to the maintenance of gastric mucosal barrier integrity and a stable microenvironment. Gastric microbial dysbiosis results in reduced microbial diversity and altered community structure, characterized by changes in the relative abundance of *HP* and abnormal enrichment of oral- and intestinal-derived microbiota, thereby compromising gastric microbial homeostasis. *HP* infection is a major driver of gastric microbial dysbiosis and mucosal barrier disruption. Its virulence factors CagA and VacA impair gastric epithelial cells, disrupt the mucus layer and tight junctions, increase mucosal permeability, and facilitate the translocation of bacterial products and carcinogenic factors into mucosal tissues, thereby inducing persistent inflammation. Furthermore, *HP* infection reduces gastric acid secretion and increases gastric pH, promoting the colonization of acid-resistant bacteria and further exacerbating microbial dysbiosis. A vicious cycle involving reduced gastric acidity, chronic inflammation, and gastric gland atrophy ultimately contributes to gastric carcinogenesis and disease progression.

Microbiota dysbiosis serves as a pivotal mechanism driving GC pathogenesis via disrupting gastric mucosal barrier integrity (Figure 5). The healthy gut microbiota is dominated by *Firmicutes*, *Bacteroidetes*, *Actinobacteria*, and *Proteobacteria*, maintaining a stable mucosal microecology. Microbiota dysbiosis is characterized by depletion of beneficial bacteria and overgrowth of pathogenic bacteria, which destroys the structural and functional integrity of the gastric mucosal barrier (Bautista et al., 2025). Patients with GC and precancerous lesions exhibit significantly reduced microbial diversity in both gastric mucosal tissues and gastric juice (Miao et al., 2022). The reduced microbial diversity and altered community structure (HP depletion or opportunistic pathogen enrichment) break the microecological balance of the stomach (He et al., 2022). The gastric mucosal barrier is the first line of defense against gastric mucosal injury, and persistent microbiota dysbiosis continuously damages barrier function. In particular, strains carrying cytotoxin-associated gene A (CagA) or vacuolating toxin A (VacA) initiate gastric mucosal barrier disruption. CagA is delivered into gastric epithelial cells via the type IV secretion system and subsequently triggers multiple intracellular signaling cascades. This process disrupts cellular signaling, thereby inducing loss of cell polarity and aberrant cell proliferation, as well as robust release of inflammatory mediators. VacA promotes intracellular organelle dysfunction, impairs epithelial cell activity, inhibits T cell function, and facilitates immune evasion. These alterations directly damage gastric epithelial cells and compromise mucosal barrier structure (Mathebela et al., 2022). A functional gastric mucosal barrier depends on intact mucus layers and tight junctions between epithelial cells. HP infection and subsequent inflammation degrade the mucus layer, disrupt tight junction protein architecture, and increase mucosal permeability. Elevated barrier permeability enables bacterial products such as LPS to interact with subepithelial immune cells and aggravate inflammatory responses. Meanwhile, carcinogens residing in the gastric lumen more readily penetrate the mucosal tissue (Huo et al., 2025). Microbiota dysbiosis further impairs gastric mucosal barrier integrity by modulating gastric acid secretion. Under physiological conditions, gastric acid eliminates most invasive pathogens and maintains gastric sterility. However, HP infection suppresses gastric acid secretion and initiates persistent chronic active gastritis. Progressive inflammation induces gastric gland atrophy and further reduces acid secretion, which elevates intragastric pH. Such microenvironmental changes facilitate the colonization of acid-resistant bacteria derived from the oral cavity and intestine, ultimately worsening microbial dysbiosis (Smolka and Schubert, 2017).

However, the evidence supporting the role of microbiota dysbiosis in disrupting the gastric mucosal barrier discussed in this section is primarily derived from *in vitro* cellular experiments and animal models. Direct causal evidence demonstrating that microbiota dysbiosis mediates gastric mucosal barrier damage and contributes to carcinogenesis in humans remains insufficient. Further validation through longitudinal cohort studies and Mendelian randomization analyses is still required.

### 3.5 DNA damage and malignant transformation of cells

**Figure 6:**
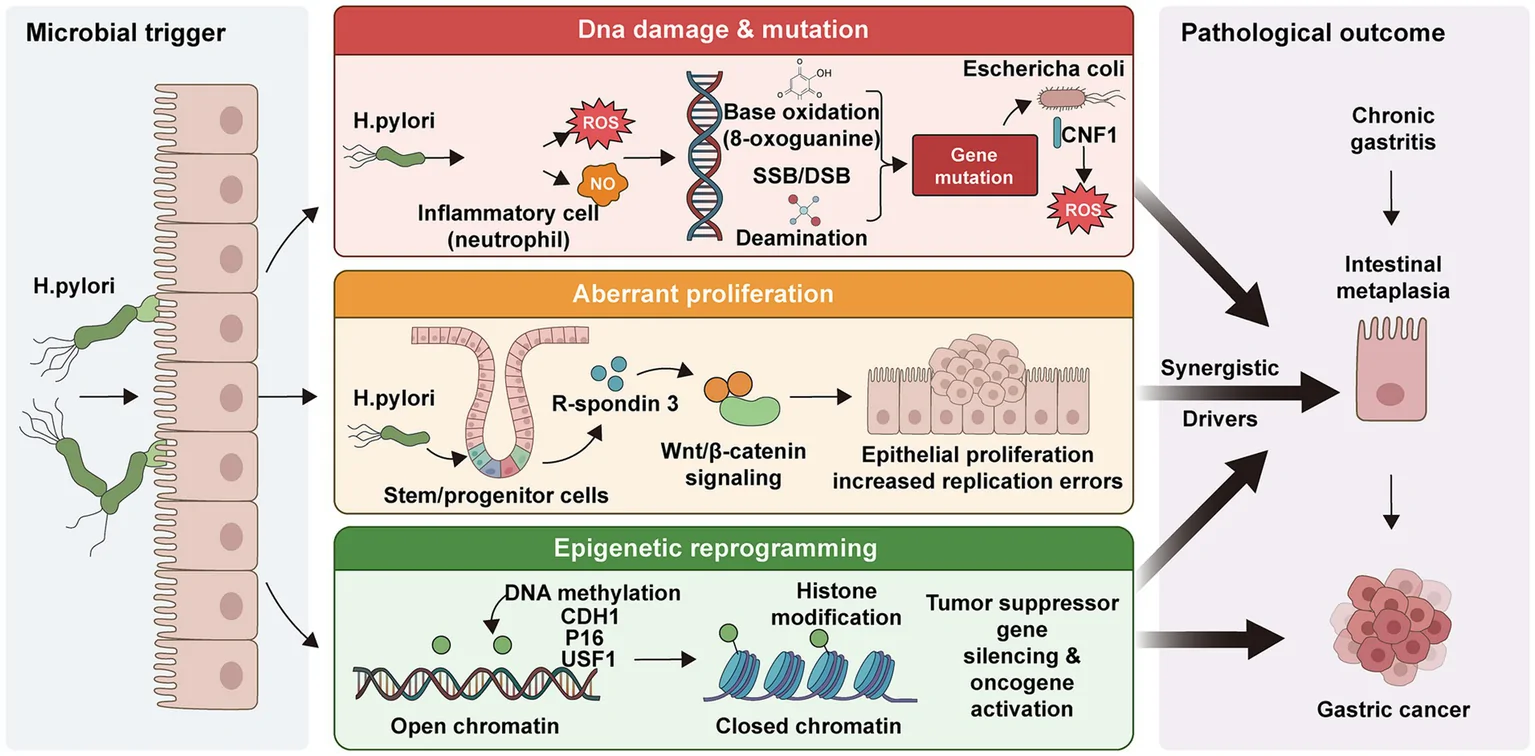
DNA damage and malignant transformation induced by gut microbiota in GC. The microbiota, particularly HP infection, contributes to the malignant transformation of gastric epithelial cells through multiple mechanisms. During HP-induced chronic inflammation, inflammatory cells such as neutrophils release ROS and reactive nitrogen species, triggering oxidative and nitrosative stress, which result in DNA base oxidation (e.g., 8-oxoguanine), DNA strand breaks, and the accumulation of genetic mutations. Moreover, bacterial virulence factors, such as *Escherichia coli* (CNF1), can enhance ROS production, exacerbate chromosomal instability, and promote DNA damage. Furthermore, HP can influence gastric stem and progenitor cells by activating the Wnt/*β*-catenin signaling pathway through induction of factors such as R-spondin 3, thereby promoting aberrant gastric epithelial cell proliferation and increasing the likelihood of DNA replication errors and mutations. In addition, microbiota-induced inflammatory environments can drive epigenetic reprogramming, including aberrant DNA methylation and histone modifications, resulting in tumor suppressor gene silencing, proto-oncogene activation, chromatin remodeling, and the acquisition of malignant phenotypes. Collectively, these mechanisms promote the multistep progression of gastric mucosal lesions from chronic gastritis and intestinal metaplasia to GC.

Microbiota dysbiosis drives DNA damage and cellular malignant transformation, representing one of the most direct mechanisms underlying microbiota-mediated gastric carcinogenesis (Figure 6). HP infection induces persistent chronic inflammation in the gastric mucosa. Inflammatory cells such as neutrophils produce ROS and nitric oxide (NO), leading to oxidative and nitrosative stress. Excessive levels of ROS can damage DNA, leading to base oxidation, including the formation of 8-oxoguanine, as well as single- and double-strand breaks. Similarly, NO gives rise to reactive nitrogen intermediates that can induce DNA deamination and base mismatches. If these lesions are not efficiently repaired, they may accumulate and ultimately result in genetic mutations (Mendes-Rocha et al., 2023). Studies have shown that cytotoxic necrosis factor 1 (CNF1) produced by *Escherichia coli* can directly induce the release of ROS in non-tumor intestinal epithelial cells, leading to chromosomal instability and DNA damage (Tozzi et al., 2025). HP can specifically target stem and progenitor cells located in the deep regions of gastric glands and induce their aberrant proliferation. For example, it may activate the Wnt/*β*-catenin signaling pathway by upregulating factors such as R-spondin 3, leading to gastric gland hyperplasia. This sustained, excessive proliferative pressure increases the likelihood of DNA replication errors, thereby fostering mutation accumulation (Wizenty and Sigal, 2025). The microbiome and its associated inflammatory environment can induce changes in the epigenetic landscape of host cells, such as DNA methylation and histone modification. For instance, HP infection can lead to hypermethylation of specific gene promoters, silencing genes like USF1. These epigenetic alterations may deactivate DNA repair genes and tumor suppressor genes or activate proto-oncogenes, thereby promoting malignant phenotypes without altering the DNA sequence (Song et al., 2025). Alterations in DNA methylation induced by HP infection are not solitary incidents; they are not only part of an extensive epigenetic reprogramming. Research indicates that HP infection is capable of inducing aberrant methylation early on during the stage of non-atrophic gastritis, without precancerous lesions. Patients with HP-positive chronic gastritis have been reported to have increased promoter methylation levels of several genes, including CDH1 (E-cadherin), GATA-5 (transcription factor), Connexin 32/43 (gap junction proteins) and p14, p16 and DAP-(death-associated protein)-kinase, in their gastric mucosae compared to uninfected patients (Tahara et al., 2009; Wang et al., 2014; Alvarez et al., 2018; Bayat et al., 2024). As the lesion develops into stage intestinal metaplasia, abnormal methylation become markedly stronger. Cells of intestinal metaplasia (IM) exhibit a unique methylation profile harboring extensive hypermethylation of tumor suppressor gene promoter CpG islands. Changes in chromatin structure caused by hypermethylation can occur, such as chromatin compaction in tumor suppressor genes. Thus, it is unstable and can increase the probability of becoming cancer cells (Takeuchi et al., 2024).

However, current evidence regarding HP-mediated DNA damage and epigenetic reprogramming is primarily derived from observational analyses of clinical specimens, *in vitro* cellular experiments, and animal models. Whether HP-induced epigenetic alterations directly promote the malignant transformation of human gastric epithelial cells remains unclear. Long-term prospective cohort studies are still required to establish the causal relationship.

### 3.6 Molecular signaling pathways and regulatory mechanisms

**Figure 7:**
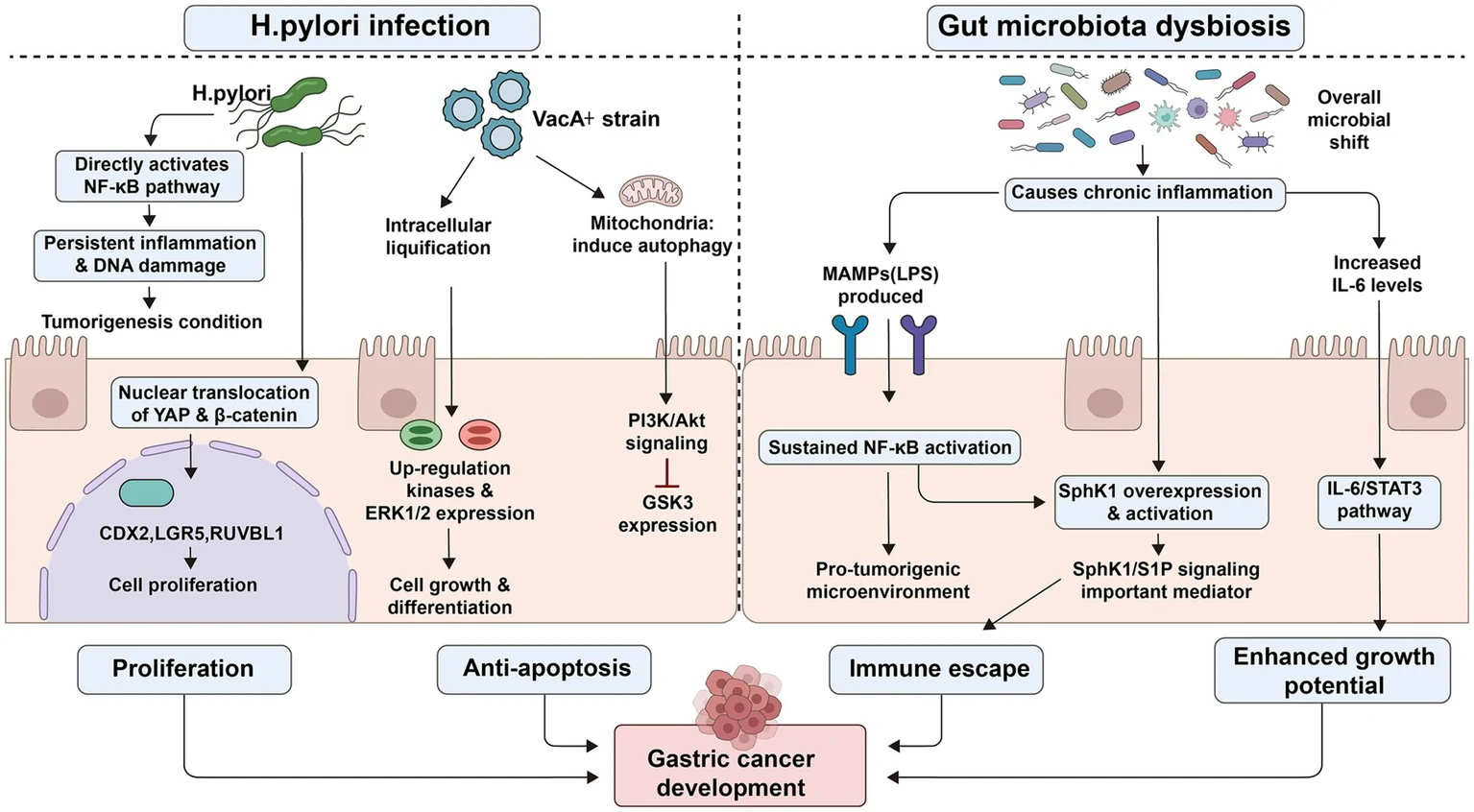
Molecular signaling pathways and regulatory mechanisms in gut microbiota-mediated GC. Intestinal microbial dysbiosis and HP infection contribute to gastric carcinogenesis through multiple molecular mechanisms. HP induces ROS production and directly activates the NF-κB signaling pathway, resulting in persistent inflammation and DNA damage. Moreover, its virulence factors, such as VacA, regulate cell proliferation, differentiation, and apoptosis resistance through the ERK1/2 and PI3K/Akt signaling pathways. In addition, HP promotes the nuclear translocation of YAP and β-catenin, activates downstream target genes including CDX2, LGR5, and RUVBL1, and drives aberrant proliferation of gastric epithelial cells. Microbial dysbiosis activates the MAMPs–NF-κB signaling axis through the production of pro-inflammatory molecules, such as LPS, thereby establishing a persistent tumor-promoting inflammatory microenvironment. Furthermore, alterations in microbial composition increase IL-6 levels, activate the IL-6/STAT3 signaling pathway, and promote tumor cell proliferation, resistance to apoptosis, and immune evasion. Dysbiosis can also activate the SphK1/S1P signaling axis, further accelerating GC progression.

Gut microbiota regulates GC initiation, progression, and treatment response through complex, interconnected molecular signaling networks (Figure 7). Moreover, many of these mechanisms influence the genesis and advancement of GC. Notably, it also regulates the tumor microenvironment that affects the treatment response. The infection of host cells by HP generates an inflammatory response associated with the production of ROS. NF-κB pathway activation by ROS stimulates persistent inflammation, generating DNA damage that favors carcinogenesis. Changes in the composition of gastric and gut microbiota (dysbiosis) may cause chronic inflammation even in the absence of HP infection. Dysregulated microbiota can result in excess production of pro-inflammatory molecules like LPS. These microbes are referred to as microbial-associated molecular patterns (MAMPs). They are recognized by pattern recognition receptors, for example, Toll-like receptors, on host immune cells. The NF-κB pathway’s continuous activation results in a milieu suitable for the instigation of tumors (Kearns, 2025). A study has demonstrated that gut microbiota dysbiosis may increase IL-6 levels, which in turn activates the STAT3 signaling pathway, thereby promoting tumor cell proliferation, resistance to apoptosis, and immune evasion, ultimately enhancing the growth potential of GC (Mohamed et al., 2025). Although this hypothesis is compelling, current evidence that gut dysbiosis promotes GC via this pathway is primarily derived from clinical correlation studies or *in vitro* experiments, and the complete *in vivo* causal chain remains to be validated using more rigorous experimental models. HP infection promotes the nuclear accumulation and transcriptional activity of YAP and *β*-catenin in gastric epithelial cells, activates target genes such as CDX2, LGR5, and RUVBL1, and stimulates cell proliferation and expansion, ultimately contributing to GC development (Li et al., 2023). HP VacA-positive strains target gastric epithelial cell mitochondria to induce cell autophagy, upregulate MAPK and ERK1/2 signaling, activate VEGF expression via the PI3K/Akt pathway, and inhibit GSK3 activity, thereby promoting tumor angiogenesis and growth (Meng et al., 2018). The sphingosine kinase (SphK)/sphingosine 1-phosphate (S1P) signaling axis plays a key role in gastrointestinal inflammation and carcinogenesis. In addition, intestinal dysbiosis may promote the overexpression and activation of SphK1, thereby facilitating GC development (Sukocheva et al., 2020). However, most current studies remain associative in nature. Future research should incorporate longitudinal cohort studies, Mendelian randomization analyses, and interventional clinical trials to further validate these findings and facilitate the transition of this field from descriptive observations toward mechanistic understanding.

In general, microbiota-driven carcinogenesis can be conceptualized as a cascade involving “barrier disruption–chronic inflammation–metabolic dysregulation–immunosuppression–DNA damage.” HP plays a key initiating role, while microbial dysbiosis synergistically amplifies carcinogenic effects. Although the aforementioned mechanisms and therapeutic strategies are promising, their translation from bench to bedside faces substantial challenges. At present, most mechanistic studies remain confined to *in vitro* systems (Qian et al., 2025) or animal models (Zhao and Yu, 2024). Although these models provide valuable causal insights, they cannot fully recapitulate the complex immune–metabolic–microbial interaction networks in humans. At the clinical level, most studies are observational in nature and therefore can only reveal associations between microbiota composition and disease status, rather than causal relationships. For example, several studies have reported alterations in gut microbial diversity and the abundance of specific taxa in patients with GC (Daly et al., 2026). However, whether these differences represent causal drivers or merely associated phenomena remains to be validated through large-scale, well-designed human interventional trials and Mendelian randomization studies. Findings from in vitro experiments and mouse models cannot be directly extrapolated to human clinical settings, which represents a major barrier to translational mechanistic research. For example, probiotics often enhance chemotherapy efficacy or immune responses in animal models; however, human clinical trials have not yielded consistently significant or reproducible results (Marashi et al., 2024). Moreover, differences in dosage, administration routes, strain selection, and host intestinal environments across experimental models can lead to effects that are difficult to replicate in humans. In summary, the gut microbiota shows substantial potential in the prevention, diagnosis, and treatment of GC. However, the field currently remains at a critical translational bottleneck between laboratory discovery and clinical application. Addressing these challenges systematically may enable microbiota-based interventions to move beyond their current role as adjunct therapies and become integral components of the comprehensive management of GC.

## 4 Therapeutic regulation strategies for gut microbiota in GC

Building upon the preceding systematic analyses of the “etiology dimension” and “injury dimension,” this section further explores the “intervention dimension” within the “three-dimensional regulatory framework” proposed in this review. Gut microbiota dysbiosis is not merely a concomitant feature of GC initiation and progression but also actively contributes to disease development by inducing multilevel pathological alterations, including persistent inflammation, immune homeostasis disruption, metabolic dysregulation, and gastric mucosal barrier impairment. Therefore, microbiota-targeted therapies should not be limited to simple modulation of microbial composition but should instead aim to restore microecological homeostasis, mitigate microbiota-mediated host injury, and enhance therapeutic efficacy. Accordingly, rather than categorizing microbiota regulation strategies solely according to therapeutic modalities, we systematically evaluate current major microecological interventions—including probiotics, fecal microbiota transplantation, antibiotic modulation, and microbial metabolite-targeted therapies—based on their regulatory effects on microbiota-associated pathological processes, as summarized in Table 2. However, most microbiota-based interventions remain at an exploratory stage, and their clinical translation is constrained by several challenges, including interindividual variability in microbiota composition, incomplete understanding of underlying mechanisms, lack of standardized efficacy evaluation criteria, and safety concerns. Therefore, by analyzing the mechanisms, advantages, limitations, and translational potential of various intervention strategies, this section aims to establish a mechanism-oriented and precision-targeted framework for evaluating microecological therapies in GC.

**Table 2:** Comparative evaluation of microbiota-targeted therapeutic strategies in GC.

Therapeutic strategy	Main mechanisms	Level of evidence	Major advantages	Major limitations	Key unanswered questions
Probiotics	Restore microbial balance; improve epithelial barrier function; regulate inflammation and immunity	Level 2–3	Good safety profile; low cost; relatively mature clinical application	Strain-specific effects; optimal dose and duration remain unclear	Which probiotic strains are most effective? Can probiotics enhance anti-cancer therapies?
FMT	Reconstruct microbial ecosystem and restore microbial function	Level 3–4	Rapid and comprehensive microbiota remodeling	Safety concerns; donor selection and standardization challenges	Which patients benefit most? How can long-term safety be ensured?
Antibiotics	Modulate specific microbial populations and eliminate pathogenic bacteria	Level 3–4	Clinically available; useful for targeted microbial control	Non-specific effects; microbiota disruption and resistance risk	How to achieve precise microbial modulation without ecological damage?
Microbial metabolites	Target microbial functional outputs to regulate immune, inflammatory, and metabolic pathways	Level 4	High mechanistic specificity; potential for precision therapy	Limited human evidence; unclear delivery and safety profiles	Which metabolites are clinically actionable biomarkers or therapeutic targets?

### 4.1 Probiotic treatment strategy

#### 4.1.1 Mechanism of action

The effects of probiotics are primarily due to their influence on the composition of a microbial community. Probiotics can reshape gut microbiota composition through competitive exclusion and the production of antimicrobial compounds. These mechanisms suppress the growth of pathogenic bacteria while facilitating the expansion of beneficial microorganisms, thereby contributing to the restoration of microbial homeostasis. In postoperative patients with GC, supplementation with compound probiotics containing *Lactobacillus plantarum*, *Lactobacillus rhamnosus*, *Lactobacillus acidophilus*, and *Bifidobacterium animalis* may help preserve the diversity and abundance of gut microbiota and prevent substantial disruption of the microbial community structure (Xiong et al., 2024). In HP eradication therapy, the concurrent use of probiotics can significantly mitigate the damage caused by antibiotics to the gut microbiota (e.g., a decrease in Firmicutes and an increase in Proteobacteria) and limit the growth of drug-resistant bacteria (Han et al., 2024). Immune regulation is a key mechanism through which probiotics exert their anti-tumor effects. Studies have shown that probiotics (e.g., *Lactobacillus* and *Bifidobacterium*) can enhance anti-tumor immunity, promote the secretion of IFN-*γ*, activate natural killer (NK) cells and CD8 + T cells, and inhibit the growth of GC tumors (Peng et al., 2025). More importantly, probiotics can enhance the efficacy of immune checkpoint inhibitors (ICIs) by reshaping the tumor immune microenvironment. Studies have shown that oral administration of *Akkermansia muciniphila* can significantly improve the efficacy of anti-PD-1 therapy in GC. The underlying mechanism involves promoting the infiltration and activation of CD8 + T cells within the tumor microenvironment (Yang et al., 2026). Probiotics also maintain intestinal homeostasis by improving mucosal barrier function. By helping to restore microbial balance, the intestinal permeability is maintained, thereby preventing the entry of endotoxins and other compounds into the bloodstream which would be harmful. This will subsequently reduce systemic inflammation and create a more favorable *in vivo* environment for immune functioning (Yang et al., 2024). Zheng et al. (2021) verified that probiotic supplementation reduces inflammation, improves immune function, restores gut microbiota homeostasis, and accelerates postoperative recovery in GC patients. Animal experiments further confirmed that compound probiotics inhibit intestinal inflammatory and permeability signaling pathways, maintaining mucosal barrier integrity and immune stability. Chronic gastrointestinal inflammation is closely coupled with GC progression, and probiotics can block GC progression by modulating intestinal immune responses and mitigating chronic inflammation. Lactobacillus-derived extracellular vesicles and cell-free supernatant downregulate HP-induced pro-inflammatory cytokines (IL-1*β*, IL-6, IL-8, TNF-*α*) and upregulate anti-inflammatory cytokines (IL-10, TGF-β), exerting robust anti-inflammatory effects (Fakharian et al., 2024).

Overall, probiotics, as a strategy for regulating the gut microbiota, demonstrate significant potential in the treatment of GC.

#### 4.1.2 Clinical research

In GC adjuvant treatment, probiotics alleviate chemotherapy and radiotherapy-related adverse reactions, improve patient immune function and quality of life, and optimize treatment tolerance. Clinical studies have shown that combined probiotic and enteral nutrition supplementation improves nutritional indicators (serum total protein, prealbumin) and immune function (IgA, IgG levels) in GC patients undergoing chemotherapy (Rahimi et al., 2021). Probiotics benefit postoperative GC recovery by reducing inflammatory responses, restoring intestinal microecology, and shortening recovery time. A prospective controlled clinical trial demonstrated that compound probiotics significantly reduce postoperative neutrophil-lymphocyte ratio (NLR) and accelerate serum albumin recovery in GC patients (*p* = 0.016) (Xiong et al., 2024). A meta-analysis further verified that probiotic supplementation shortens the first postoperative defecation time by an average of 11.27 h, reduces hospital stay by 0.94 days, and lowers postoperative infection complication risks (RR = 0.46) (Ye et al., 2023). As an adjuvant therapy for HP eradication, probiotics reduce HP recurrence and antibiotic-related side effects. In bismuth quadruple therapy (BQT), supplementary probiotics (especially *Saccharomyces boulardii* and multi-strain formulations) significantly improve HP eradication rates (OR = 1.49),with superior efficacy in East Asian populations and amoxicillin-clarithromycin combination regimens (Liu et al., 2025).

Overall, probiotics, as a microbiota-modulating strategy, exhibit considerable potential in GC management. However, the therapeutic efficacy of probiotics is influenced not only by strain-specific characteristics but also by multiple host-related factors, including baseline microbiota composition, tumor stage, immune status, and concurrent treatment regimens. Consequently, different patients may exhibit distinct or even opposing responses to the same probiotic intervention. For example, in patients with advanced GC characterized by baseline microbial dysbiosis and enrichment of Streptococcus and Enterobacteriaceae, simple supplementation with Lactobacillus or Bifidobacterium may fail to achieve effective colonization and could potentially interact with pre-existing tumor-promoting microbial communities, thereby enhancing pro-inflammatory metabolite production and exacerbating systemic inflammation (Djukovic et al., 2022). Therefore, future clinical applications should incorporate patient-specific microbiota characteristics and functional metabolic profiles to enable precise probiotic selection, rather than adopting a “one strain fits all” treatment strategy.

### 4.2 Treatment strategy of fecal microbiota transplantation

#### 4.2.1 Mechanism of action

FMT is a novel microecological therapy that reconstructs intestinal microecological homeostasis by transplanting functional microbiota from healthy donors to patients. The core therapeutic mechanism of FMT in GC is the reconstruction of balanced gut microbiota. GC patients suffer from disease- and treatment-induced intestinal dysbiosis, characterized by depleted beneficial bacteria and overgrown pathogens. FMT replenishes deficient beneficial microbes and suppresses pathogenic bacterial proliferation, restoring normal intestinal microecological structure and function (Xu et al., 2024). Immune regulation is a key anti-tumor mechanism of FMT. FMT-mediated microbial remodeling normalizes the interaction between MAMPs and the NF-κB signaling pathway, enhances host tumor immune surveillance, and reverses immunosuppression (Mohamed et al., 2025). FMT reshapes the tumor immune microenvironment to improve immunotherapy efficacy. In GC patients receiving combined FMT and anti-PD-1 therapy, clinical benefits are closely associated with the colonization of donor-derived immunogenic bacteria and peripheral immune cell activation, indicating that FMT reverses tumor immunosuppression and enhances ICIs therapeutic efficacy (Zhang et al., 2026). FMT also regulates intestinal microbial drug metabolism, optimizes the pharmacokinetic characteristics of chemotherapeutic drugs, improves treatment efficacy, and reduces toxic side effects (Wu M. et al., 2024). In addition, FMT alleviates GC-related chronic intestinal inflammation by inhibiting NLRP3 inflammasome activation and upregulating anti-inflammatory IL-10 expression (Yao et al., 2022).

#### 4.2.2 Clinical research

Fecal microbiota transplantation can influence GC progression by modulating the gut microbiota and its metabolites. For instance, the *Blautia coccoides* strain and its derivative, 5Z-dodecenoic acid, exhibited a significant protective effect in a chronic psychological stress-induced GC model. The reduction of this bacterial strain is associated with the malignant phenotype of GC, including enhanced glycolytic activity and increased invasiveness. Supplementation with *Blautia coccoides* or its metabolites may reverse these effects. The underlying mechanism involves the direct inhibition of the oncogenic protein RIOK2 and disruption of its interaction with BYSL (Zhao et al., 2025). Currently, clinical research on FMT in GC remains in its early stages. However, it has progressed from exploring feasibility and safety to preliminary efficacy evaluation. A Phase I study involving immune checkpoint inhibitor (ICI)-refractory advanced gastrointestinal cancer (including 8 cases of GC) demonstrated that the combination of FMT (administered via oral capsules) and anti-PD-1 therapy (nivolumab) was well tolerated, with no serious adverse events, thus providing preliminary evidence for the safety and feasibility of this combined regimen (Zhang et al., 2026). Another Phase I-II study evaluates the safety of FMT following antibiotic pretreatment in patients with unresectable advanced or recurrent esophageal/GC who are scheduled to receive ICI-containing regimens, with its primary endpoint focusing on the incidence of dose-limiting toxicity. Initiated in June 2024, this ongoing study has enrolled 7 patients as of August 2025 (Yoshinami et al., 2025). However, it should be noted that these findings are preliminary, and the full results of this ongoing trial are awaited to confirm the safety and efficacy of FMT in this patient population.

Restoration of intestinal microecological balance represents the fundamental mechanism underlying the therapeutic effects of fecal microbiota transplantation (FMT). However, FMT does not simply restore a so-called “healthy microbiota”; rather, its efficacy is highly dependent on donor microbiota characteristics, the recipient’s intestinal ecological environment, and host immune status. Because patients differ substantially in their ability to support the colonization of exogenous microorganisms, FMT may result in variable therapeutic benefits or even treatment failure. Microbial communities that demonstrate efficacy in animal models may fail to engraft in human recipients due to colonization resistance or may even activate pro-inflammatory pathways through the introduction of specific opportunistic pathogens. Moreover, transplantation of donor microbiota enriched with tumor-promoting microbial species or carcinogenic metabolite-producing communities may exacerbate intestinal dysbiosis and accelerate tumor progression. Therefore, FMT should not be developed as a “standardized preparation,” and efficacy prediction should precede clinical validation. Future FMT research should prioritize the identification of optimal donors, prediction of microbial functional capacity, and assessment of recipient suitability to improve therapeutic success rates.

### 4.3 Antibiotic treatment strategies

#### 4.3.1 The dual mechanism of antibiotics

Antibiotics play a dual role in the treatment of GC: they can offer therapeutic benefits by eradicating pathogenic bacteria, but they may also negatively affect the normal microbiota. HP infection is the most significant risk factor for GC, and antibiotics can substantially reduce the risk by eliminating this pathogen (Chen H. et al., 2025). HP infection upregulates pro-inflammatory factors such as IL-1β and IL-6, and certain antibiotics can lower the levels of these inflammatory mediators, thereby mitigating the associated inflammatory response (Li et al., 2024). In some instances, the gut microbiome enhances the effectiveness of chemotherapy owing to antibiotics. Studies have found that antibiotics suppress H2-producing bacteria, alter the tumor microenvironment, and increase the sensitivity of therapy (Chen Z. et al., 2025). Furthermore, some antibiotics based on nanoparticles can penetrate HP biofilms, thus increasing drug permeability (Yu et al., 2023). It is also important to consider the risks associated with antibiotic use. Antibiotics can promote the emergence and dissemination of drug-resistant bacteria. Studies have reported a global increase in resistance to clarithromycin, metronidazole, and fluoroquinolones (Rokkas and Graham, 2026). In addition to this, antibiotics may disrupt gut microbiota composition by reducing bacterial diversity and quantity. This may favor the selection of more resistant bacterial strains (Fiorani et al., 2023).

#### 4.3.2 Risks and challenges of antibiotic therapy

Antimicrobial resistance poses a serious challenge to antibiotic therapy. The resistance of HP over the time has gradually augmeneted to the most commonly prescribed antibiotics, basically clarithromycin and metronidazole. The emergence of drug resistance results in a decrease in the rate of eradication and an increase in treatment failure. Accordingly, treatment selections should be based on local drug resistance surveillance data. The potential for side effects is a significant concern in antibiotic treatment. Side effects from ET may include the gastrointestinal disturbance, allergic reactions, and impairment of liver and kidney function, etc. The use of antimicrobials poses the long-term risk of microbial imbalance. Antibiotics can change the composition of the gut microbiota to reduce the amount of useful bacteria or it can increase harmful microorganisms. The risk of complications may increase, such as diarrhea, constipation, and malnutrition.

In general, antibiotics represent an important component of GC management; however, their bidirectional effects on the microbiota require highly individualized and judicious clinical application. Appropriate antibiotic use can eradicate pathogenic microorganisms and alleviate inflammation in patients with HP infection. In contrast, unnecessary broad-spectrum antibiotic exposure may disrupt intestinal homeostasis, exacerbate microbial dysbiosis, and impair antitumor immunity. Particularly in the era of immunotherapy, broad-spectrum antibiotic administration may compromise the efficacy of ICIs by reducing microbial diversity and attenuating immune activation. Therefore, optimizing antibiotic stewardship, together with microbiota-preserving strategies such as probiotics and gut microbiota restoration, represents a critical approach to balancing the dual effects of antibiotics and improving GC prevention and treatment outcomes.

### 4.4 Microbial metabolite therapy strategy

#### 4.4.1 Mechanism of action

SCFAs are the important molecules formed as the result fermentation of the dietary fibers by intestinal microbes mainly acetic acid propanoic acid butanoic acid. SCFAs seem to be critical regulators of GC treatment. Immune modulation may be the main mechanism by which they exert anti-tumor effects. Microbially derived immunometabolites can influence tumor biology by modulating the phenotype and function of immune cells. In particular, these metabolites regulate the secretion of immunosuppressive cytokines (Qiu et al., 2020). Moreover, SCFAs block histone deacetylase expression and increase immune cells’ actions. They bind to their receptors, namely GPR41 and GPR43. And they particularly affect effector T cells (Wang et al., 2023). Epigenetic regulation is a key mechanism through which SCFAs exert their effects. Among these, butyric acid is a potent inhibitor of HDAC. By inhibiting HDAC activity, SCFAs can regulate gene expression, influencing cell proliferation, differentiation, and apoptosis. Accumulating evidence indicates that butyric acid promotes macrophage differentiation and enhances antibacterial activity via HDAC3 inhibition, which regulates glycolysis and autophagy to modulate tumorigenesis and tumor suppression (Dong et al., 2023). Additionally, anti-inflammatory effects represent a significant function of SCFAs. SCFAs promote IL-22 production in CD4^+^ T cells by activating GPR41 and inhibiting histone deacetylase activity, thereby suppressing inflammatory responses (Yang et al., 2020). In addition to SCFAs, intestinal microbes produce a variety of other key metabolites that also play significant roles in GC treatment. For example, secondary bile acids, such as deoxycholic acid (DCA), are generated by intestinal microorganisms through 7α-dehydroxylation of primary bile acids and exert a broad range of effects on host metabolism. DCA may play a critical role in cancer development by modulating multiple signaling pathways, including the EGFR–MAPK and *β*-catenin signaling pathways and the p53 pathway (Zhao et al., 2023). Additionally, intestinal microorganisms can metabolize tryptophan to produce a series of indole compounds. These metabolites influence intestinal barrier function, immune response, and inflammation levels by activating aromatic hydrocarbon receptors and other signaling pathways, thereby modulating the therapeutic effects in GC (Gou et al., 2024).

#### 4.4.2 Clinical research

Certain microbial metabolites can directly promote tumorigenesis. For example, *Streptococcus* species like *Streptococcus anginosus* synthesize methionine through the action of the metabolic enzyme metE gene tax enzymes, promoting GC progression *in vitro* and *in vivo* (Zhou et al., 2026). According to clinical studies, the impact of SCFA mixture (APB) used in combination with Doxorubicin (Dox) harbors low toxicity and offers a suitable proposal to enhance chemotherapy efficacy against GC by inducing apoptosis, disturbing redox balance and decreasing drug resistance (Eladwy et al., 2025a). Studies have shown that an increase in specific bacteria, such as *Escherichia coli*-*Shigella*, is associated with elevated levels of metabolites like N-acetylcadaverine and *ε*-caprolactam. These changes are closely linked to a high SIRI, which reflects poor prognosis (Zou et al., 2025). Zhu et al. (2024) demonstrated that traditional Chinese medicine compounds, such as Jianpi Yangzheng Decoction, can inhibit the formation of a pre-metastatic microenvironment in GC by remodeling the gut microbiota and restoring SCFA levels, thereby playing an anti-metastatic role. In addition, this combination downregulates the oncogen TNS4, inhibits the NF-κB signaling pathway, and blocks the platelet activation network, significantly augmenting the anti-proliferative effect, while also resulting in an anticancer effect. The theory built after going through all these findings will help SCFAs function as chemotherapy adjuvants (Eladwy et al., 2024; Eladwy et al., 2025b).

Currently, microbiota-targeted regulatory strategies are at different stages of development, and their clinical value depends not simply on their ability to alter microbial composition but on whether they can achieve precise modulation tailored to specific disease stages, therapeutic contexts, and individual microbiome characteristics. Therefore, based on a comprehensive evaluation of existing evidence, it is necessary to establish a clinical scenario-based framework for selecting microecological interventions to facilitate the translation of research findings into clinical practice, as summarized in Table 3.

**Table 3:** Potential clinical applications of microbiota-targeted interventions in GC.

Clinical scenario	Potential intervention strategies	Therapeutic rationale	Current evidence level
Early-stage GC or gastric precancerous lesions with HP infection	Probiotics combined with dietary modulation and HP eradication	Restore microbial balance and reduce inflammation	Moderate
Advanced GC receiving immunotherapy	Probiotics, FMT, or microbiota-based combination strategies	Enhance anti-tumor immunity and improve immunotherapy response	Preliminary
GC patients undergoing chemotherapy	Probiotics and microbiota-supportive interventions	Reduce treatment-related toxicity and maintain microbial stability	Moderate
Postoperative GC patients	Probiotics and nutritional intervention	Promote microbiota recovery and improve gastrointestinal function	Moderate
Refractory or treatment-resistant GC	FMT or microbial metabolite-based therapy	Remodel microbial ecology and target microbiota-derived functions	Low

It should be noted that the effects of microbial metabolites on tumors are highly dose- and context-dependent. The concentration-dependent dual effects of metabolites represent a major obstacle to their clinical translation: simple supplementation with exogenous SCFAs or bile acid preparations cannot precisely control local metabolite concentrations and may inadvertently shift their effects from tumor suppression to tumor promotion. Moreover, orally administered metabolites are often degraded in the upper gastrointestinal tract and may fail to reach the gastric tumor microenvironment at effective concentrations. Systemic administration may disrupt whole-body metabolic homeostasis and induce unpredictable effects on tumor regulation. This complexity makes it challenging to achieve consistent therapeutic outcomes in clinical trials involving standardized metabolite dosing, highlighting the need for patient-specific strategies based on baseline metabolic profiles. Therefore, the direct development of microbial metabolites as therapeutic agents requires comprehensive characterization of their therapeutic windows, target cell populations, and host metabolic contexts. Future research should prioritize functional profiling of metabolite networks rather than simply measuring changes in individual metabolite levels.

In summary, among microecological interventions, probiotics represent an adjuvant therapy with a relatively well-established evidence base, clear short-term safety, and emerging clinical applicability. The highest level of evidence derives from a single-center randomized controlled trial (Zheng et al., 2019).

However, important limitations remain: the therapeutic efficacy of probiotics varies substantially among individuals, and outcomes are influenced by multiple factors, including strain composition, dosage, duration of intervention, baseline gut microbiota profiles, and concurrent chemoradiotherapy. In addition, the long-term safety of continuous supplementation has not been fully established. FMT is theoretically advantageous because it may restore a complete set of missing commensal microbiota, increase the abundance of beneficial butyrate-producing bacteria, and reduce systemic chronic inflammation. However, it remains at an exploratory stage, lacking randomized controlled trials and robust anti-tumor clinical endpoints. Moreover, there is no standardized protocol for donor selection or transplantation procedures, microbial engraftment is often unstable, and potential long-term safety risks, including cross-infection, remain a concern. At present, FMT should be considered an investigational approach restricted to clinical research settings rather than routine clinical practice. Microbial metabolites represent the lowest level of evidence among these interventions, with most findings derived from *in vitro* cell-based experiments and animal models. Significant translational barriers limit their clinical application. For instance, oral SCFAs are rapidly degraded in the upper gastrointestinal tract and therefore fail to achieve effective concentrations within the gastric tumor microenvironment. Suitable human-targeted delivery systems remain under development, placing this approach at the greatest distance from clinical translation. In addition, antibiotic interventions exhibit a clear duality in their potential benefits and risks. Targeted antibiotic therapy is recommended only for the primary prevention of HP–positive atrophy and intestinal metaplasia. In contrast, the inappropriate use of broad-spectrum antibiotics in advanced GC may disrupt intestinal homeostasis and attenuate the efficacy of immunotherapy. These limitations underscore important therapeutic constraints, indicating that antibiotic use must be strictly restricted to appropriate clinical indications.

Future research should shift its focus from “verifying whether an intervention is effective” to “predicting who will benefit, when intervention should be implemented, how non-responders can be identified in advance, and how therapeutic strategies can be dynamically optimized throughout the treatment process.” Achieving this goal requires systematic investigation of the three-way causal interactions among the microbiota, immune system, and tumor biology at the basic research level, rather than merely describing correlations. At the clinical research level, efforts should progress from single-center exploratory studies toward multicenter, long-term follow-up, microbiota-guided randomized controlled trials. At the translational level, priorities include establishing standardized donor screening systems, developing targeted metabolite delivery platforms, and implementing real-time microbiota monitoring technologies. With the gradual refinement of this integrated framework, gut microbiota-based therapeutic regulation is expected to overcome the current challenge of “abundant mechanistic insights but limited therapeutic strategies,” become incorporated into comprehensive GC prevention and treatment throughout the disease course, and provide patients with low-toxicity, personalized, and dynamically adjustable intervention strategies.

## 5 Clinical translation of gut microbiota-based therapy for GC: from theoretical exploration to precision application

Although gut microbiota-targeted intervention strategies have demonstrated considerable potential in GC treatment, substantial challenges remain before their successful clinical translation. Future research should move beyond merely exploring associations between microbial compositional changes and therapeutic outcomes and should instead focus on establishing a systematic translational framework that integrates precise patient stratification, optimization of intervention strategies, efficacy evaluation systems, biomarker identification, and safety management.

First, more precise patient selection and stratification strategies should be implemented in future clinical studies. Currently, most microbiota-related clinical studies have enrolled highly heterogeneous populations of patients with GC, which may contribute to substantial variability in treatment responses and limit the generalizability of research findings. Therefore, future clinical trials should stratify patients according to key factors influencing gut microbiota composition and therapeutic responses, including tumor stage, prior treatment regimens, HP infection status, antibiotic exposure history, dietary patterns, immune status, and baseline microbiota characteristics. For example, patients with GC receiving ICIs may represent an important target population for microbiota-based interventions, as accumulating evidence indicates that gut microbes can modulate immunotherapy efficacy by regulating antitumor immune responses. Accordingly, future prospective clinical studies should perform baseline microbiota profiling before intervention to identify potential responders and improve the precision of microbiota-based therapeutic strategies.

Second, clinical trial designs should establish more comprehensive and standardized efficacy evaluation systems. Currently, clinical studies evaluating GC therapies primarily rely on conventional clinical endpoints, including objective response rate, progression-free survival, overall survival, and treatment-related adverse events. However, for microbiota-modulating therapies, traditional efficacy indicators alone may be insufficient to fully capture their underlying mechanisms and therapeutic benefits. Therefore, future studies should incorporate microbiota-related parameters into efficacy assessment frameworks, including alterations in microbial diversity, enrichment of beneficial microorganisms, depletion of potentially pathogenic species, changes in microbial metabolite profiles, and immune-related indicators (e.g., tumor-infiltrating lymphocyte activity and peripheral immune cell composition). Furthermore, integrating multi-omics approaches, including metagenomics, metabolomics, transcriptomics, and immunomics, may enable comprehensive characterization of how microbiota-based interventions influence GC initiation, progression, and therapeutic responses.

Thirdly, priority should be given to the identification and validation of predictive and pharmacodynamic biomarkers. The successful implementation of microbiota-based therapies in precision medicine largely depends on the ability to accurately predict which patients are likely to derive clinical benefit. Currently, potential candidate biomarkers include the abundance of specific microbial taxa, microbial functional gene signatures, the capacity to produce key metabolites such as SCFAs, and features of the host immune microenvironment. Certain immunomodulatory bacterial taxa may serve as potential biomarkers for predicting responses to immunotherapy. For example, beneficial commensal bacteria, including *Faecalibacterium prausnitzii* and *Akkermansia muciniphila*, have been associated with enhanced anti-tumor immune responses and improved efficacy of immune checkpoint inhibitors (Peddireddi et al., 2025). Therefore, future studies should establish and validate integrated multi-marker predictive models through large-scale, multicenter clinical trials to enable patient stratification, minimize ineffective treatments, and improve clinical outcomes.

Finally, a unified standardization framework and long-term safety evaluation system should be established before the widespread clinical implementation of microbiota-based therapies. Because the gut microbiota is influenced by multiple factors, including geographic region, dietary habits, medication exposure, sequencing technologies, and individual genetic backgrounds, substantial limitations exist in the comparability of findings across different studies. Therefore, future clinical research should establish standardized protocols for microbial sample collection, storage, sequencing, analysis, and data processing. Meanwhile, rigorous quality control systems should be developed for emerging microbiota-based therapeutic approaches, including FMT, engineered probiotics, next-generation probiotics, and synthetic microbial consortia. These systems should encompass donor screening, pathogen detection, preparation standardization, and long-term follow-up monitoring to minimize potential risks associated with infectious transmission, ecological disruption of the microbiota, and abnormal immune responses.

Overall, the future development of gut microbiota-based therapies for GC should gradually transition from traditional “empirical microbiota modulation” toward “precision microbiome-based therapy.” By establishing patient-specific stratification systems, optimizing microbiota intervention strategies, incorporating multidimensional efficacy evaluation frameworks, and developing reliable predictive biomarkers, microbiota-based therapeutic approaches are expected to advance from experimental investigation toward clinical application.

## 6 Conclusion

This review systematically summarizes the characteristic alterations of gut microbiota in GC patients, elaborates on the multi-dimensional mechanisms by which gut microbiota dysbiosis drives GC pathogenesis, and concludes current microbiota-targeted therapeutic strategies for GC. Accumulating evidence confirms that gut microbiota plays a pivotal role in GC initiation and progression via immune dysregulation, chronic inflammatory activation, carcinogenic metabolite secretion, and gastric mucosal barrier disruption. Restoring intestinal microecological balance has become a promising novel therapeutic direction for GC.

At present, microbiota-regulatory strategies, including probiotics, fecal microbiota transplantation, antibiotic therapy, and microbial metabolites, have gradually entered the stage of clinical exploration, demonstrating preliminary efficacy and safety in several small-scale studies. However, substantial interindividual variability in microbiota composition remains a major challenge, and current research is still limited by unresolved causal relationships, inconsistent therapeutic efficacy, lack of standardized intervention protocols, and insufficient long-term safety assessments.

Over the next 5–10 years, research on GC microecology is expected to gradually transition from mechanistic exploration toward precision medicine applications. First, large-scale, multicenter longitudinal cohort studies combined with Mendelian randomization analyses are needed to further elucidate the causal relationships between microbiota dysbiosis and GC initiation and progression, thereby distinguishing microbial drivers from disease-associated alterations. Second, standardized microbiota-based therapeutic frameworks should be established, including donor screening criteria for FMT, microbiota preparation procedures, therapeutic dosing strategies, and safety monitoring protocols, to facilitate the standardized implementation of microecological interventions. In addition, future studies should focus on predictive and pharmacodynamic biomarkers by integrating microbial composition, functional characteristics, metabolites, and host immune profiles to develop multidimensional models for patient stratification and therapeutic response prediction. Finally, large-scale randomized controlled trials and long-term follow-up studies are required to systematically evaluate the safety, durability, and clinical applicability of microbiota-targeted interventions, as well as their potential for combination with existing therapeutic modalities, such as immunotherapy.

Overall, gut microbiota modulation provides a novel theoretical framework and promising clinical opportunities for the precision prevention and treatment of GC. With continued advances in multi-omics technologies, artificial intelligence-based analytical approaches, and clinical research platforms, individualized intervention strategies guided by microbiome characteristics are expected to become an integral component of comprehensive GC management and drive the evolution of treatment paradigms toward greater precision and personalization.
